# Steering the Absorption Configuration of Intermediates over Pd-Based Electrocatalysts toward Efficient and Stable CO_2_ Reduction

**DOI:** 10.1021/jacs.4c14253

**Published:** 2025-01-24

**Authors:** Shuting Wei, Yanchao Xu, Tao Song, Hao Dai, Fan Li, Xin Gao, Yanjie Zhai, Shanhe Gong, Rui Li, Xiao Zhang, Kangcheung Chan

**Affiliations:** † Department of Industrial and Systems Engineering, The Hong Kong Polytechnic University, Kowloon 999077, Hong Kong, China; ‡ Department of Chemistry, Southern University of Science and Technology, Shenzhen 518055, China; § Department of Mechanical Engineering, The Hong Kong Polytechnic University, Kowloon 999077, Hong Kong, China; ∥ Institute of Clean Energy, Yangtze River Delta Research Institute, Northwestern Polytechnical University, Xi’an 710072, China; ⊥ Research Institute for Advanced Manufacturing, The Hong Kong Polytechnic University, Kowloon 999077, Hong Kong, China

## Abstract

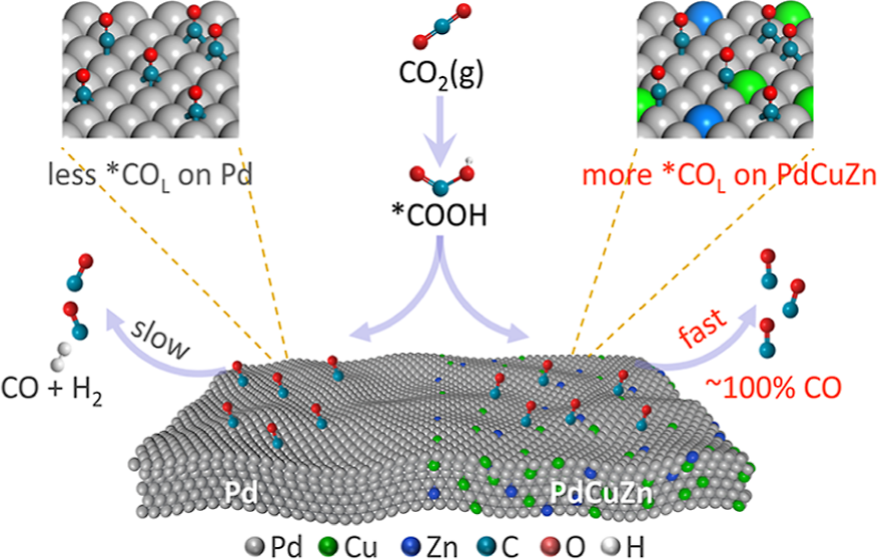

Palladium (Pd) catalysts are promising for electrochemical reduction of CO_2_ to CO but often can be deactivated by poisoning owing to the strong affinity of *CO on Pd sites. Theoretical investigations reveal that different configurations of *CO endow specific adsorption energies, thereby dictating the final performances. Here, a regulatory strategy toward *CO absorption configurations is proposed to alleviate CO poisoning by simultaneously incorporating Cu and Zn atoms into ultrathin Pd nanosheets (NSs). As-prepared PdCuZn NSs can catalyze CO production at a wide potential window (−0.28 to −0.78 V vs RHE) and achieve a maximum FE_CO_ of 96% at −0.35 V. Impressively, it exhibits stable CO production of 100 h under ∼95% FE_CO_ with no decay. Combined results from X-ray analysis, in situ spectroscopy, and theoretical simulations suggest that the codoping strategy not only optimizes the electronic structure of Pd but also weakens the binding strengths of *CO and increases the proportion of weak-binding linear *CO absorption configuration on catalysts’ surfaces. Such targeted adoption of weakly bound configurations abates the energy barrier of *CO desorption and facilitates CO production. This work confers a useful design tactic toward Pd-based electrocatalysts, codoping for steering adsorption configuration to achieve highly selective and stable CO_2_-to-CO conversion.

## 1 Introduction

Electrochemical CO_2_ reduction into value-added chemicals and fuels powered by renewable electricity represents a promising approach to alleviate the carbon emission issue, balance the future carbon cycle, and eventually establish a sustainable energy economy.^1−4^ Multiple products ranging from C_1_ to C_3+_ oxygenates and hydrocarbons can be obtained via the CO_2_ reduction reaction (CO_2_RR).^5−11^ Among various products, CO has attracted widespread attention due to its sizable market and economic feasibility.^12−14^ To date, many metal candidates (such as Cu, Ag, Au, Zn) have been studied for CO_2_-to-CO conversion, in which Pd exhibits potential for CO formation because of its facile CO_2_ absorption and *COOH formation.^15,16^ Unfortunately, the strong *CO binding on the Pd surface makes the subsequent *CO desorption a sluggish step and results in severe poisoning of active sites, finally deactivating the CO_2_-to-CO conversion within hours.^17,18^ The development of efficient Pd-catalysts with a suitable *CO binding energy still needs substantial improvements.

**Scheme 1:**
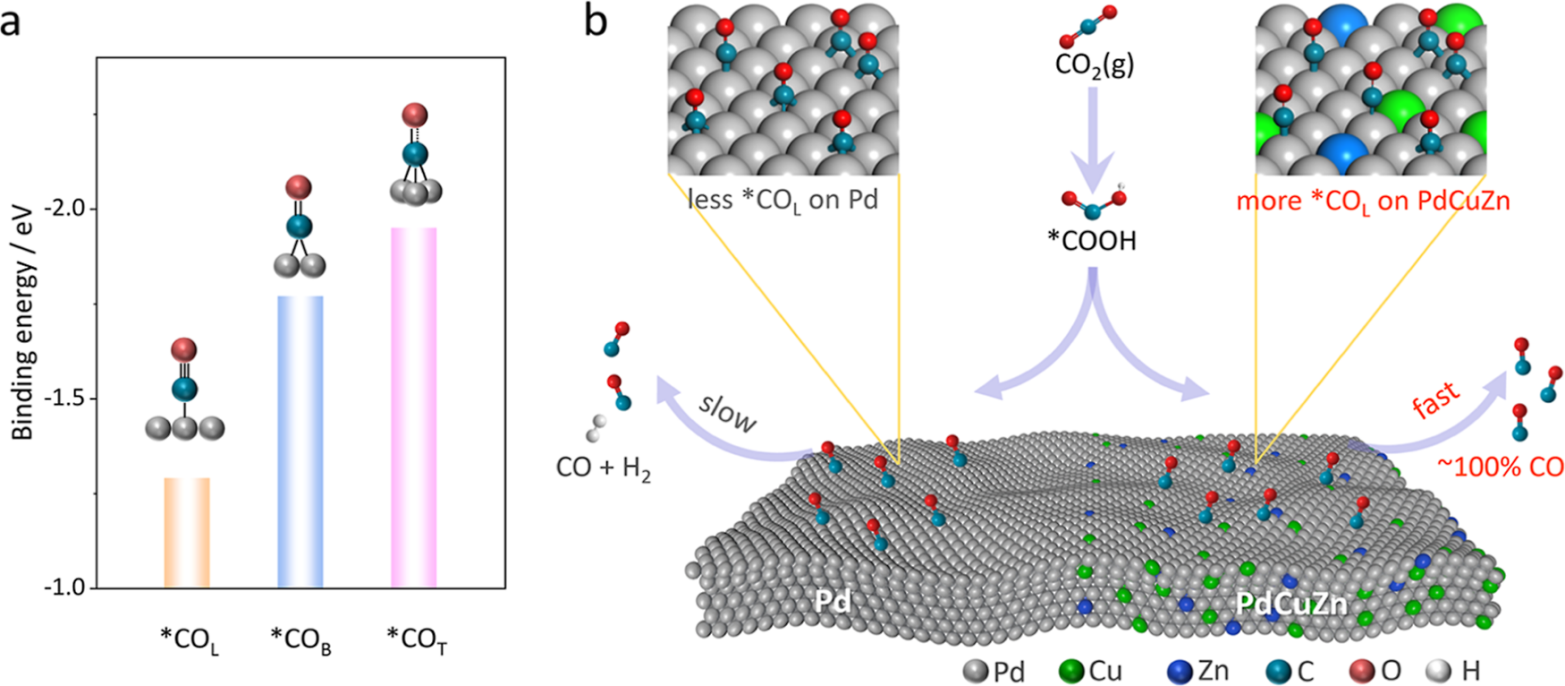
(a) Specific Binding Energies of *CO with Different Configurations on the Pd(111) Surface; (b) Schematic Illustration Showing the Impact of Cu/Zn Incorporation on the CO_2_-to-CO Process

The binding strength of reaction intermediates is determined by the specific adsorption behaviors.^19,20^ Theoretical studies have shown that there are three types of *CO configurations including linearly bound (CO_L_), bridge bound (CO_B_), and triple bound (CO_T_) on metal surfaces.^21^ As summarized in Scheme 1a, the binding strength of *CO on the Pd(111) surface gives a sequence of *CO_L_ (−1.29 eV) < *CO_B_ (−1.77 eV) < *CO_T_ (−1.95 eV),^22^ which makes *CO_L_ favorable for the subsequent desorption from Pd sites. The specific absorption behavior of reaction intermediates can be influenced by the electronic structure of active sites,^23,24^ which provides possibilities to tailor the adsorption configurations via subtle electronic regulations. Alloying represents a powerful tool to modify the catalyst’s electronic structure,^25,26^ in which the shifts of metal d-band are closely related to the binding affinity of intermediates on active sites.^27,28^ Such regulating tactics have been widely utilized as the general design principles of bimetallic Pd alloys with refined electronic structures, resulting in high selectivity of Pd-based alloys for CO_2_RR.^29−32^ However, the impact of Pd alloying toward detailed adsorption behaviors, especially the influence of alloying Pd with multiple metals toward specific adsorption configurations of key reaction intermediates, is scarcely explored, which is believed to be the intrinsic factor in determining the final activities.

Two-dimensional ultrathin metals show a large surface area and expose the active sites to a great extent, which provides an ideal platform to study the influences of alloying-induced electronic coupling and strain effects on adsorption behaviors.^33−35^ Cu and Zn have different electronic structures and CO adsorption ability from Pd. Introducing Cu with suitable *CO binding interaction into Pd helps to reduce the energy barrier between the intermediate and substrate.^30,36^ Zn is located on the weakly bound side of the volcano apex, which is conducive to CO desorption.^37^ The simultaneous introduction of Cu and Zn into Pd is expected to greatly optimize the electronic structure and alleviate the absorption strength of CO on Pd. Therefore, in this work, the ultrathin Pd alloy nanosheets (NSs) are designed and fabricated for boosting electrocatalytic CO_2_-to-CO conversion by Cu and Zn coincorporation. The as-prepared PdCuZn NSs with few atomic layers exhibit remarkable activity and selectivity toward CO production from CO_2_RR, which shows a maximum Faradaic efficiency (FE_CO_) of 96% at −0.35 V (vs RHE), far more superior than that of PdCu (80% FE_CO_) and PdZn (65% FE_CO_) alloys. Thanks to the strong electronic coupling between multiple components and ultrathin structures, PdCuZn NSs also exhibit excellent long-term stability of 100 h electrolysis without current and selectivity loss. The membrane electrode assembly test of as-prepared PdCuZn NSs also shows stable FE_CO_ (∼80%) under −200 mA cm^–2^ for 45 h, suggesting their promising potentials in terms of practical conditions. Experimental results manifest that simultaneous codoping of Cu and Zn optimizes the catalyst’s electronic structure and lowers the d-band center, which effectively regulates the binding affinity of reaction intermediates on the Pd surface. In situ electrochemical spectroscopy analysis and density functional theory simulations reveal that the introduction of Cu and Zn not only steers the adsorption configurations of *CO on the Pd sites but also weakens the bonding strength of *CO on Pd sites. As illustrated in Scheme 1b, by the codoping of Cu and Zn, the key *CO intermediates on PdCuZn are more likely to adopt the linear *CO_L_ configuration (with weak CO binding) instead of the dominant triple-bonded configuration (with strong CO binding) on pure Pd, which finally weakens the *CO binding on Pd sites. The alleviated CO poisoning of Pd expedites the rate-determining *CO desorption process and finally facilitates the selective conversion of the CO_2_RR to CO products.

## 2 Results and Discussion

**Figure 1:**
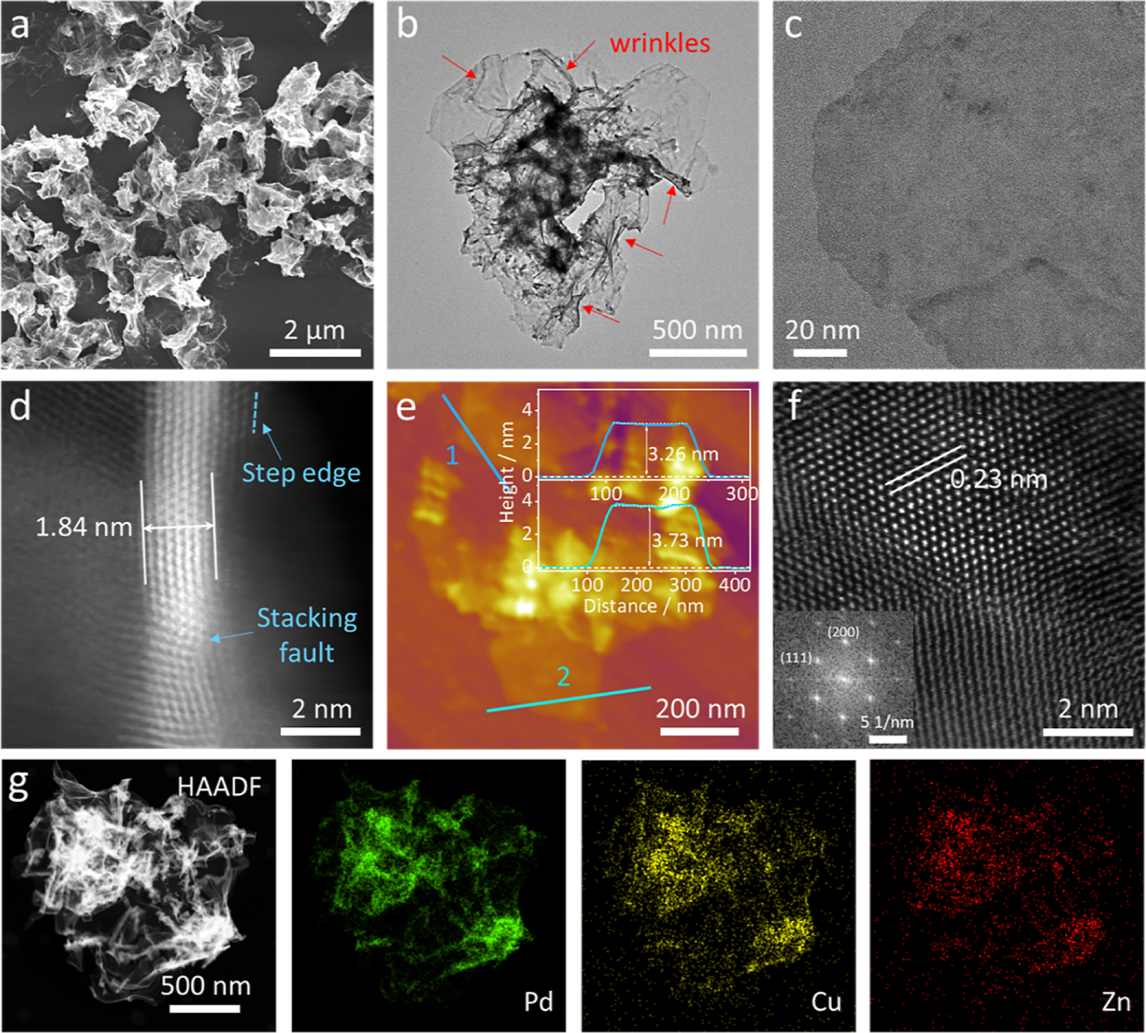
Morphology and structure characterization of PdCuZn NSs. (a) SEM image, (b,c) TEM images, (d) HAADF-STEM image, (e) AFM image (inset: the corresponding height profiles of the PdCuZn NSs), (f) high-resolution HAADF-STEM image, and (g) EDS elemental mappings.

The structural information on Cu- and Zn-codoped Pd alloys (PdCuZn) was first characterized by scanning electron microscopy (SEM) and transmission electron microscopy (TEM). In Figure 1a, the SEM image of as-prepared PdCuZn alloys exhibits irregular and undulant NS morphology with sizes ranging from one to several micrometers. The low-magnification TEM image in Figure 1b shows abundant wrinkles and corrugations on the NSs. The corrugated characteristic implies a flexible and ultrathin nature of the PdCuZn NSs, which is confirmed by the low contrast of single NS in the high-magnification TEM image (Figure 1c). The side view of a selected NS in a high-angle annular dark-field scanning transmission electron microscopy (HAADF-STEM) image (Figure 1d) shows that the thickness is as low as 1.84 nm, which is equivalent to about eight atomic layers. The thickness of the NSs is further determined to be ∼3–4 nm by atomic force microscopy (AFM) in Figure 1e. The height profiles of two randomly selected regions show specific thicknesses of 3.26 and 3.73 nm, respectively, which are thicker than those measured by an electron microscope, probably due to the possible stacking and the adsorbed capping agent on the NSs.^38^ As shown in Figure 1f, the aberration-corrected HAADF-STEM image shows clear lattice fringes with an interplanar spacing of 0.23 nm, corresponding to 1/3 (422) of the face-centered cubic (fcc) Pd, manifesting the emergence of stacking faults parallel to the basal (111) planes associated with the Pd NSs.^39,40^ The fast Fourier transform (FFT) pattern (inset of Figure 1f) also manifests the fcc crystal structure with a predominant (111) plane of the as-resulting PdCuZn NSs. Elemental mapping analysis in Figure 1g reveals the homogeneous distribution of Pd, Cu, and Zn throughout the selected NSs, indicating their alloy character. The composition ratio of Pd/Cu/Zn in PdCuZn NSs is calculated to be 3.99/1.96/1.00 by inductively coupled plasma-optical emission spectroscopy (ICP-OES) (Table S1). In addition, the pure Pd NSs, Cu-doped Pd NSs (defined as PdCu NSs), and Zn-doped Pd NSs (defined as PdZn NSs) were also prepared as references to investigate the influences of multiple components toward the CO_2_RR. Their detailed structural analysis can be found in the Supporting Information in Figures S1–S3.

**Figure 2:**
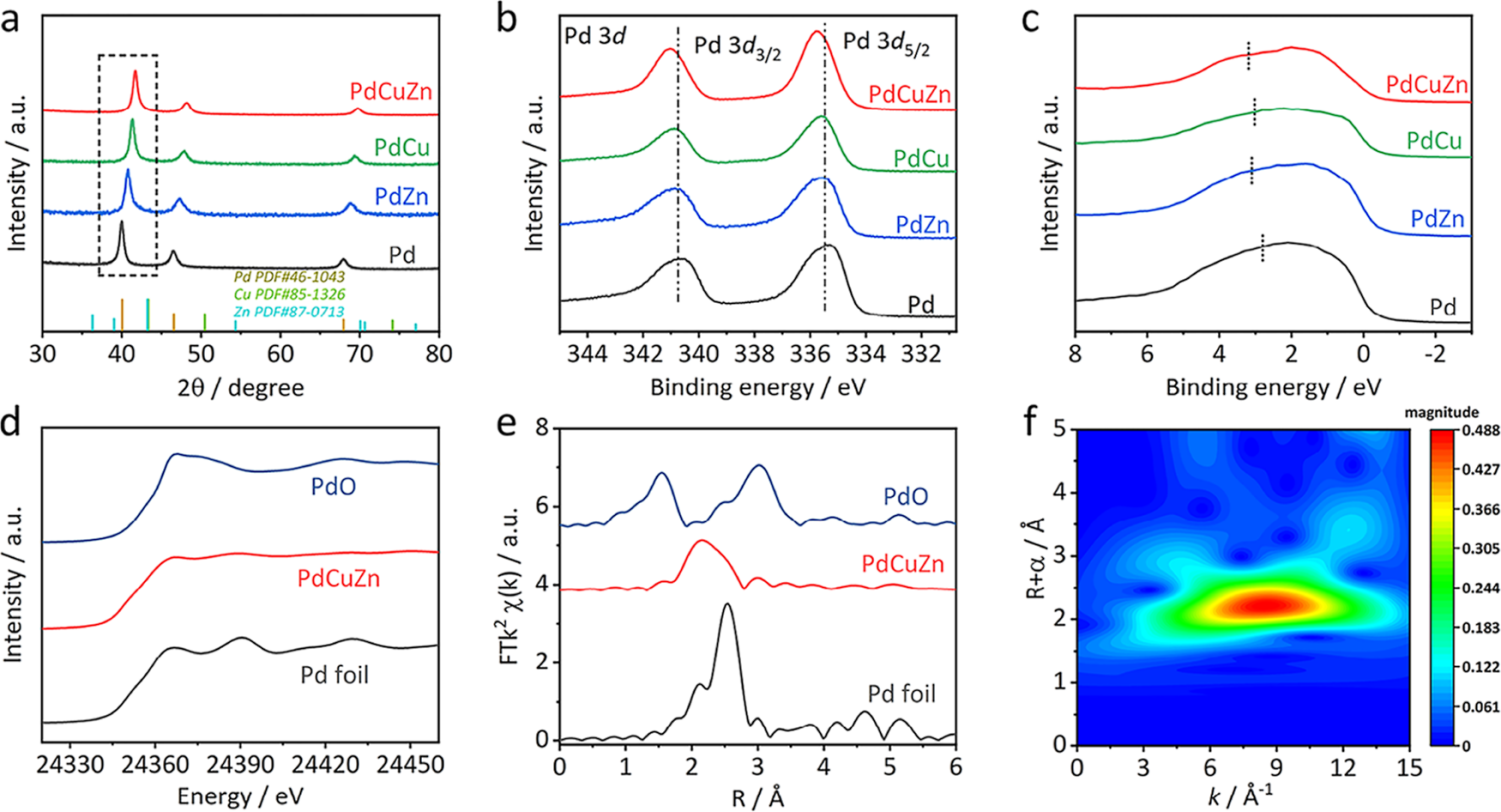
Crystal and electronic structure analysis. (a) XRD patterns, (b) Pd 3d XPS spectra, and (c) the valence band spectra of different samples. (d) XANES spectra, (e) FT-EXAFS spectra of the Pd K-edge region, and (f) WT-EXAFS pattern at the Pd K-edge for PdCuZn NSs.

The crystal structure of as-prepared samples was analyzed by X-ray diffraction (XRD). As exhibited in Figure 2a, pure Pd NSs show three typical diffraction peaks at 39.9°, 46.5°, and 67.9°, assigned to (111), (200), and (220) planes of Pd with a fcc structure. The PdCuZn, PdCu, and PdZn NSs display the same fcc structure as Pd NSs, in which their diffraction peaks shift to higher angles compared with pure Pd, confirming their alloy nature. Notably, the diffraction peaks of PdCuZn NSs demonstrate a more positive shift when compared with PdCu and PdZn NSs, which is attributed to the coincorporation of Cu and Zn with a small atom radius. Such codoping results in stronger compressive strain in the Pd lattice than that of bimetallic counterparts, which probably changes the electronic structure of Pd and affects the binding of absorbed species on active sites more efficiently. X-ray photoelectron spectroscopy (XPS) was performed to further study the effects of Cu/Zn doping on the electronic structure of the Pd sites. As shown in Figure S4, the Pd 3d XPS spectrum of PdCuZn NSs displays two peaks, corresponding to the spin–orbit double peak of Pd 3d_5/2_ and Pd 3d_3/2_, respectively. The peaks at 335.77 and 341.02 eV are attributed to Pd^0^ 3d_5/2_ and Pd^0^ 3d_3/2_, while the peaks at 337.15 and 342.90 eV belong to Pd^2+^ 3d_5/2_ and Pd^2+^ 3d_3/2_, respectively. In the comparison of PdCu, PdZn, and Pd NSs, the Pd 3d XPS spectrum of PdCuZn NSs shifts to a higher binding energy (Figure 2b). The positive shift of Pd binding energy indicates that the d-band center is far away from the Fermi level,^31,41^ which is beneficial for diminishing the binding affinity of key intermediates and improving the catalytic performance for the CO_2_RR. For the Cu 2p XPS spectrum of PdCuZn NSs, the peaks at 931.85 and 951.60 eV belong to Cu^0^ 2p_5/2_ and Cu^0^ 2p_3/2_, respectively, while the peaks at 933.85 and 953.60 eV correspond to Cu^2+^ 2p_5/2_ and Cu^2+^ 2p_3/2_, respectively. The Cu 2p XPS spectrum of PdCuZn NSs shifts to a lower binding energy than that of PdCu NSs. A similar phenomenon occurs in Zn 2p XPS spectra (Figure S5). These results demonstrate that the simultaneous introduction of bimetallic atoms greatly modifies the electronic structure of Pd. The d-band center is quantitatively determined by the XPS valence band spectra. As shown in Figure 2c, the d-band centers of PdCuZn, PdCu, and PdZn NSs are estimated to be 3.20, 3.07, and 3.14 eV, respectively. All alloyed Pd NSs shift away from the Fermi level when compared with pure Pd (2.84 eV), among which PdCuZn NSs have the lowest d-band center. The most dramatic downshift over the d-band might contribute to reducing the absorption between the active sites and reactants or key intermediates, thus dictating the catalytic performance of catalysts.^42−44^

The valence states and coordination circumstances of PdCuZn NSs were further investigated by X-ray absorption spectroscopy (XAS). The X-ray absorption near-edge structure (XANES) of each metal element in PdCuZn NSs is shown in Figures 2d and S6a,b, respectively. The absorption spectra of Pd, Cu, and Zn K-edges of PdCuZn NSs coincide well with those of metal foils, indicating that Pd, Cu, and Zn in PdCuZn NSs mainly exist in the metallic valence state, which is in accordance with the XPS results. The Fourier transform EXAFS (FT-EXAFS) spectrum of the Pd K-edge region for PdCuZn NSs in Figure 2e exhibits a main peak at 2.20 Å, assigned to the nearest Pd–M bond (M = Pd, Cu, or Zn). The bond distance of the Pd–M bond is shorter than that of the Pd–Pd bond (2.54 Å) of Pd foil, signifying the existence of compressive strain in the lattice due to the doping of Cu and Zn atoms, in good agreement with the XRD results. According to previous reports, the compressive strain will lower the d-band center and in turn weaken the binding toward reaction intermediates.^45,46^ The FT-EXAFS spectrum of Pd in PdCuZn NSs also shows an obvious decrease in the intensity of the Pd–M bond. Such a decrease suggests that the coordination number (CN) of Pd atoms in PdCuZn NSs is reduced after Cu/Zn codoping, which leads to the coordinatively unsaturated status of Pd sites. A main peak at 2.45 Å in the FT-EXAFS spectra of the Cu K-edge and a prominent peak of 2.39 Å in the FT-EXAFS spectra of the Zn K-edge in PdCuZn NSs are also observed in Figure S6c,d, assigned to the Cu–M bond and Zn–M bond, respectively. The wavelet transform-EXAFS (WT-EXAFS) pattern of each metal element in PdCuZn NSs was obtained to further provide coordinate information in *k* and *R* space, as shown in Figures 2f and S6e,f. The WT-EXAFS spectrum at the Pd K-edge for PdCuZn NSs shows that a maximum intensity at ∼8.45 Å^–1^ in the *k*-space is resolved at 2.20 Å in the *R*-space, which is ascribed to Pd–M coordination. In addition, a maximum signal of Cu–M coordination and Zn–M coordination is located at 9.75 and 6.95 Å^–1^, respectively, demonstrating the existence of Pd–Cu/Zn heteroatomic bonds. The FT-EXAFS spectra were fitted using the ARTEMIS module to gain an in-depth understanding of the local structure of the PdCuZn catalysts. The fitting results are shown in Figure S7, and the structural parameters are listed in Table S2. The EXAFS fitting curve of the Pd K-edge reveals that the coordination shell of Pd contains Pd–Cu and Pd–M backscattering paths. The PdCuZn NSs exhibit the Pd–Cu bond with a CN of 1.89 and the Pd–M bond with a CN of 8.64, respectively, which are lower than those in bulk Pd foil (12). The Pd–M bond length is 2.67 Å, which is smaller than the Pd–Pd bond length of bulk materials (2.74 Å), suggesting the presence of compressive strain caused by alloying effects. The EXAFS fitting curve of the Cu K-edge exhibits the Cu–Cu bond with a CN of 1.57 and a bond length of 2.55 Å, slightly larger than the value of bulk Cu (2.53 Å). The EXAFS fitting curve of the Zn K-edge shows that the CN of the Zn–Cu bond is 1.09, and the bond length is 2.57 A, which is less than the value of the metal Zn (2.66 Å). The above analysis results demonstrate the coexistence of lattice strain effects and coordinatively unsaturated Pd sites in PdCuZn NSs after the simultaneous incorporation of Cu and Zn into ultrathin Pd layers, which is conducive to enhancing the catalytic activity.

**Figure 3:**
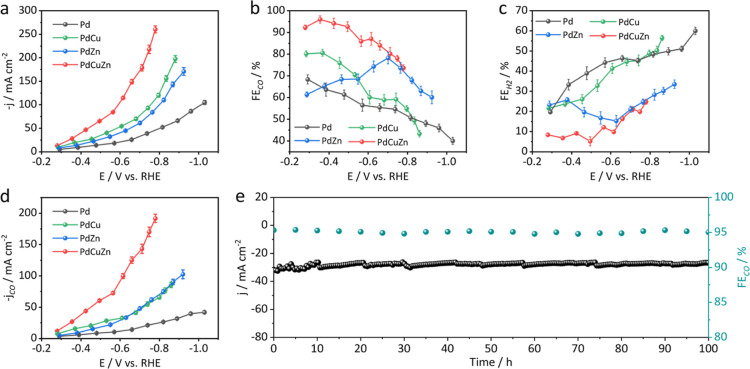
Electrochemical CO_2_RR performance. (a) Total current density of PdCuZn, PdCu, PdZn, and Pd NSs for the CO_2_RR. (b) FE_CO_ of PdCuZn, PdCu, PdZn, and Pd NSs for the CO_2_RR. (c) FE_H2_ of PdCuZn, PdCu, PdZn, and Pd NSs for the CO_2_RR. (d) Comparison of CO partial current densities of CO at various potentials. (e) The total current density and FE_CO_ of the PdCuZn NSs over long-term electrolysis.

The electrochemical performances of as-prepared samples for the CO_2_RR were measured in a three-compartment flow cell (Figure S8). Different compositions of Cu and Zn in Pd-based electrocatalysts have an important effect on the electrochemical performance of the CO_2_RR performance. As-prepared PdCuZn NSs with a Pd/Cu/Zn atomic ratio of 3.99/1.96/1.00 were identified to be the optimum candidate for the CO_2_RR due to their high CO selectivity and current density (Figure S9). In addition, PdCu, PdZn, and pure Pd were also tested under the same conditions for comparison. Linear sweep voltammetry (LSV) curves were first recorded under Ar and CO_2_ atmospheres. As shown in Figure S10, the LSV curves of PdCuZn, PdCu, and PdZn NSs tested under a CO_2_ atmosphere exhibit larger current density and lower onset potential than those tested under an Ar atmosphere in the measured potential range, which suggests that PdCuZn, PdCu, and PdZn NSs are very sensitive to the CO_2_RR. Particularly, PdCuZn NSs possess the best catalytic activity under a CO_2_ atmosphere among all tested catalysts, indicating the excellent intrinsic CO_2_RR activity of PdCuZn NSs. In addition, the LSV curve of Pd NSs displays a lower current density under a CO_2_ atmosphere than under an Ar atmosphere, demonstrating that the HER might prevail over the CO_2_RR on pure Pd NSs. The total current density of PdCuZn, PdCu, PdZn, and Pd NSs for the CO_2_RR is summarized in Figure 3a. PdCuZn NSs display the highest current density (260.4 mA cm^–2^), notably higher than that of PdCu (197.5 mA cm^–2^) and PdZn (170.7 mA cm^–2^), indicating that the cointroduction of Cu and Zn can significantly improve the catalytic activity. Compared with Cu/Zn-doped Pd catalysts, pure Pd exhibits a much lower total current density (104.8 mA cm^–2^). The FEs of CO_2_RR products of PdCuZn, PdCu, PdZn, and Pd NSs under different potentials are shown in Figure S11. Only two gas products (CO and H_2_) were identified by gas chromatography (GC), and no liquid products were detected by nuclear magnetic resonance (NMR) after electrolysis (Figures S12 and S13). The FEs of CO_2_RR products heavily depend on the applied potentials, and a much higher current density is observed on PdCuZn than the other three catalysts at all potentials. The efficiency diagrams of the CO products of these four catalysts at different potentials are shown in Figure 3b. The PdCuZn NSs exhibit higher FE_CO_ than the other three catalysts under the whole applied potential. Remarkably, the FE_CO_ of PdCuZn NSs exceeds 92% over the potential range of −0.25 to −0.50 V. A maximum FE_CO_ of 96% is obtained at −0.35 V, corresponding to an overpotential of 240 mV, which is comparable to the performance of Pd-based catalysts reported under the same test conditions (Table S3). Moreover, the FE_CO_ exceeds 80% in the range from −0.28 to −0.71 V. By contrast, the FE_CO_ of PdCu, PdZn, and Pd NSs is less than 81% over the applied potentials. The FEs of H_2_ of these catalysts under different potentials are shown in Figure 3c. As-prepared PdCuZn NSs display lower FE_H2_ than the other three catalysts under the whole applied potentials. Figure 3d shows the plots of partial current density (*j*_CO_) versus the applied potentials of the four catalysts. The *j*_CO_ of all four catalysts presents an increasing trend as the cathodic potential. Notably, the maximum j_CO_ of PdCuZn NSs is 191.65 mA cm^–2^, much larger than that of PdCu (85.52 mA cm^–2^), PdZn (102.56 mA cm^–2^), and Pd NSs (41.92 mA cm^–2^).

The electrochemically active surface area (ECSA) is also an important descriptor in dictating the catalytic activity. Therefore, the ECSAs of catalysts were evaluated from the integrated charge of Pd oxide reduction by measuring cyclic voltammetry (CV) curves, as shown in Figure S14. The ECSAs were then calculated to be 40.30, 99.05, 165.24, and 170.00 cm^2^ for PdCuZn, PdCu, PdZn, and Pd NSs, respectively. The ECSA and mass-normalized current densities of the four catalysts are shown in Figure S15 to evaluate the intrinsic activity. The Pd-based alloys display enhanced specific and mass activities compared to pure Pd NSs. In particular, PdCuZn NSs have the highest specific activity and mass activity among Pd-based alloys. Specifically, PdCuZn NSs show a specific activity of 4.76 mA cm^–2^ and a mass activity of 277.44 A g^–1^_Pd_ at −0.78 V. Electrochemical impedance spectroscopy (EIS) was performed to study charge-transfer dynamics in the electrochemical process.^47^ As shown in Figure S16, PdCuZn NSs show a smaller ohmic resistance (*R*_s_) of 1.9 Ω and a charge-transfer resistance (*R*_ct_) of 3.0 Ω than other Pd-based catalysts, indicating that the coincorporation of Cu and Zn brings in good conductivity and fast charge-transfer dynamics for the electrocatalytic process. The long-term stability of the PdCuZn catalyst was also measured using the chronoamperometry technique method, during which the product analysis from the CO_2_RR via GC was done every 5 h. As shown in Figure 3e, the current density displays a negligible deterioration even after 100 h of continuous testing. More remarkably, the FE_CO_ remained so well at ∼95% throughout the entire electrolysis. We further evaluated the long-term durability at −200 mA cm^–2^ using a membrane electrode assembly (MEA) electrolyzer. Specifically, the “PdCuZn/GDL || Ni foam” system exhibits good durability for CO_2_RR electrolysis at −200 mA cm^–2^, which maintains the FE_CO_ at ∼80% for 45 h with negligible selectivity loss (Figure S17), indicating its potential toward practical applications. Such outstanding performances of long-term electrolysis might be attributed to the strong electronic coupling between multicompetent and unique ultrathin structures, which makes PdCuZn NSs a promising catalyst for practical application toward CO_2_-to-CO conversion. Furthermore, PdCuZn after long-term electrolysis was also characterized by XRD, TEM, and XPS (Figures S18–S20). The XRD pattern shows that PdCuZn still maintains a fcc phase. The intensity of the diffraction peak is weaker than before electrolysis, which is caused by the detachment of some catalysts from GDL during the electrolysis process. The TEM image shows that PdCuZn still retains the flexible, corrugated, and ultrathin NS morphology after electrolysis. The HRTEM image also indicates that PdCuZn still maintains a fcc structure with exposed (111) planes. In addition, the EDS elemental mappings manifest that the Pd, Cu, and Zn elements are uniformly distributed throughout the NSs. The XPS spectra indicate that the composition and valence state remain almost unchanged after electrolysis. These results confirm the robust structural stability of the PdCuZn NSs.

**Figure 4:**
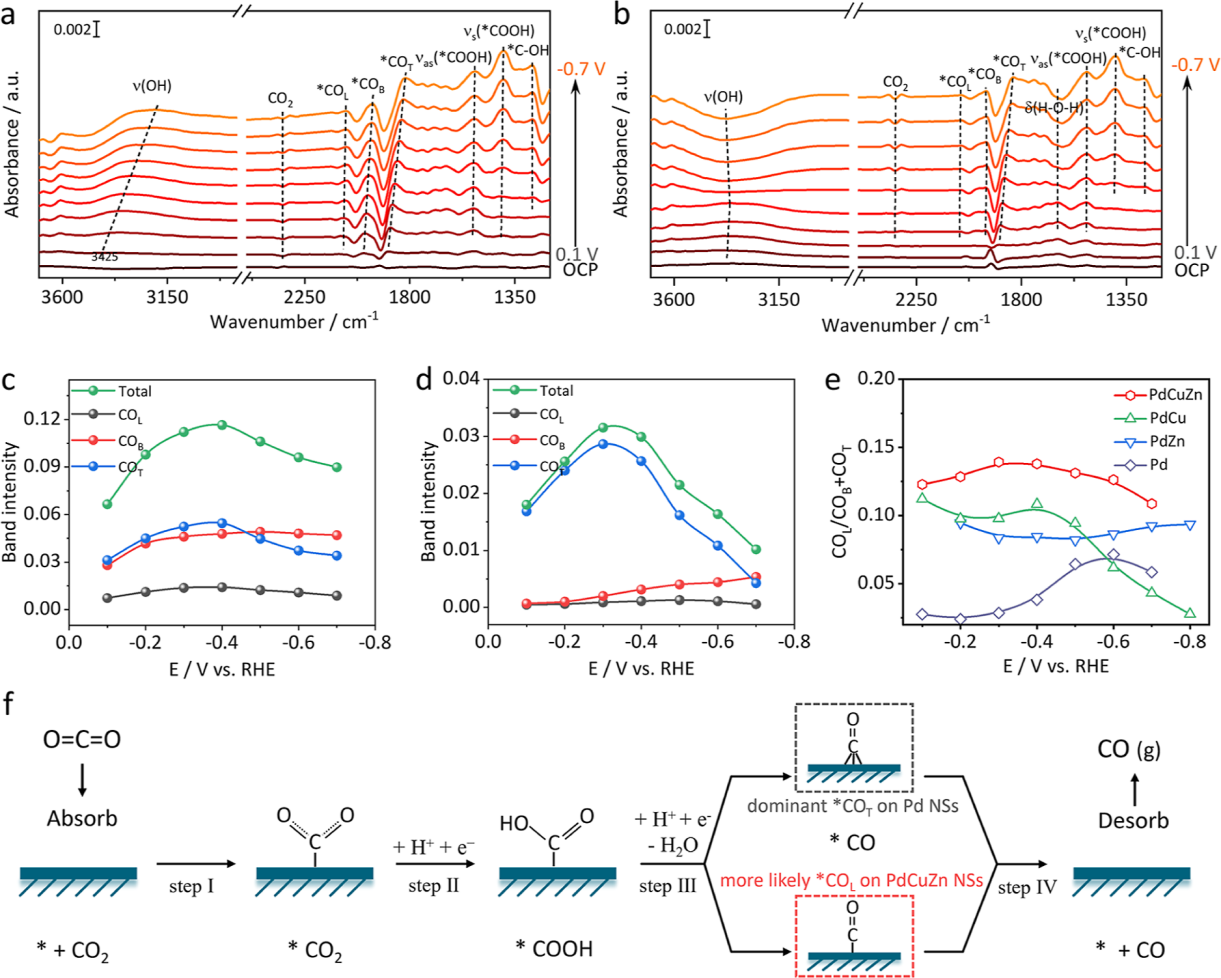
Mechanism insights. In situ ATR-FTIR spectroscopy of (a) PdCuZn and (b) Pd NSs with CO_2_ purging. Potential-dependent *CO band intensity of (c) PdCuZn and (d) Pd NSs. (e) *CO_L_ to *CO_B_ + *CO_T_ band intensity ratio. (f) Proposed CO_2_RR to the CO process over PdCuZn and pure Pd NSs.

In order to gain a deeper understanding of the enhanced activity and CO selectivity upon Cu/Zn incorporation, in situ attenuated total reflectance-Fourier transform infrared (ATR-FTIR) absorption spectroscopy was carried out to monitor the evolution of intermediates on the PdCuZn and Pd NSs under operating conditions (Figure S21). Figure 4a,b shows in situ ATR-FTIR spectra of PdCuZn and Pd NSs under a CO_2_ atmosphere, which were recorded during the cathodic scanning process in a CO_2_-saturated 0.2 M KHCO_3_ solution from 0.1 V to −0.7 V. The characteristic bands vary versus bias potentials, which corresponds to the adsorption behavior of reagents and intermediates during the CO_2_RR process. A downward band appeared at 2343 cm^–1^ which belongs to CO_2_ consumption in the solution.^48^ Two positive-going bands at 1410 and 1531 cm^–1^ were observed at 0.1 V, which are assigned to symmetric and asymmetric stretching mode of O–C–O from *COO^–^, respectively,^49^ indicating that the CO_2_ activation is facile on the PdCuZn catalyst. A positive-going band appeared at 1280 cm^–1^ and was ascribed to the OH-deformation of the *COOH intermediate.^49^ As the cathode potential increases, the characteristic bands at ∼2071, 1974, and 1855 cm^–1^ appear at 0 V, ascribed to linearly bound *CO_L_, bridge-bound *CO_B_, and triple-bound *CO_T_, respectively, implying a rapid conversion from *COOH to *CO.^21,50^ The *CO band intensity becomes stronger, and the bands shift to lower wavenumbers with the increase of potential. Such a blue shift of *CO bands is induced by Stark effects, which is derived from the changes of the interfacial electric field.^51−53^ The characteristic bands appear in the 3200–3400 cm^–1^ region, corresponding to the stretching vibration of OH from adsorbed H_2_O,^54^ which is derived from the water generated during the CO_2_RR process or from the water in the solution. For in situ ATR-FTIR spectra of Pd NSs, the CO_2_ peak (2343 cm^–1^) appears at −0.1 V, which is more negative than that of the CO_2_ peak in PdCuZn NSs, indicating that the CO_2_ adsorption on PdCuZn NSs is much easier than on Pd NSs. In addition, the vibration peak intensities of *CO bands (2059 cm^–1^ for *CO_L_, 1951 cm^–1^ for *CO_B_, and 1836 cm^–1^ for *CO_T_) on Pd NSs are weaker than those of PdCuZn NSs between 0 and −0.7 V, indicating PdCuZn NSs are more favorable for CO_2_ activation and subsequent conversion toward CO compared with Pd NSs. Particularly, the characteristic bands appear at ∼1645 cm^–1^ in the spectra of Pd NSs, which is assigned to the H–O–H bending vibration mode of H_2_O. Meanwhile, the O–H stretching vibration of H_2_O also appears at ∼3378 cm^–1^.^55,56^ Remarkably, at lower potentials, the vibrational peaks of H_2_O are upward, which is attributed to H_2_O produced by the CO_2_RR process. However, the vibration peaks of H_2_O turn downward as the potential increases, which represents the consumption of H_2_O to produce H_2_ in the electrolyte. This transition from H_2_O production to H_2_O consumption indicates that the CO_2_RR is dominant on Pd NSs at lower potentials, while the activity of competing H_2_O decomposition exceeds that of the CO_2_RR at higher potentials. This phenomenon is consistent with the results of electrochemical CO_2_RR testing on Pd NSs. In situ ATR-FTIR spectra of PdCu and PdZn NSs are shown in Figure S22a,b, and PdCu and PdZn exhibit typical vibrational peaks similar to PdCuZn and Pd NSs. The CO_2_ peaks of PdCu and PdZn appear at −0.1 V. With the appearance of the characteristic CO_2_ peak, the vibration peaks of *CO bands (*CO_L_, *CO_B_, and *CO_T_) also begin to appear. However, only *CO_L_ peaks are obviously observed at −0.1 V for PdZn. As the potential increases, *CO_B_ and *CO_T_ bands appear, and the peak intensities gradually increase. In addition, a control experiment of electrolysis under an Ar-saturated 0.2 M KHCO_3_ solution was further performed to investigate whether the CO_2_ reduction activity of PdCuZn NSs comes from CO_2_ molecules dissolved in solution or from CO_2_ molecules released by bicarbonate ions. As shown in Figure S23, the *CO characteristic band also occurred in the 2000–2100 cm^–1^ region under an Ar atmosphere, but the onset potential of *CO is higher, and the intensity of *CO is rather weaker than that in a CO_2_ atmosphere. This weak *CO signal may be derived from the reduction of CO_2_ molecules released by bicarbonate ions.^21,57^ The above analysis demonstrates that solution-phase CO_2_ dominates the CO_2_RR of PdCuZn NSs.

The configuration of adsorbed *CO (CO_L_, CO_B_, or CO_T_) on the catalyst surface plays critical roles in determining its binding affinity and desorption rate, in turn dictating the overall performance. Therefore, potential-dependent plots of *CO_L_, *CO_B_, and *CO_T_ areas in the total *CO bands were performed by integration to visualize the adsorption states of *CO on the catalyst surface, as shown in Figures 4c,d and S22c,d. First, PdCuZn NSs exhibit much stronger *CO band intensity compared with those of Pd, PdCu, and PdZn NSs under all selected potentials, indicating that the *CO intermediate more favorably appears on PdCuZn NSs than on other catalysts. The potential-dependent plots of PdCuZn NSs show that obvious *CO intensities begin at −0.1 V and then quickly increase to −0.4 V, reaching a maximum value of 0.12. Importantly, *CO_B_ and *CO_T_ show a fair intensity under the entire potential window, far higher than the *CO_L_ intensity. However, for Pd NSs, the *CO_T_ dominates and its intensity is much higher than that of *CO_L_ and *CO_B_ under the entire potential window (Figure 4d). The potential-dependent integration analysis highlights that PdCuZn NSs are more likely to adopt *CO_L_ or *CO_B_ configuration, while pure Pd NSs prefer *CO_T_ as the dominant configuration when catalyzing the CO_2_ to CO process. As a key intermediate for producing CO, the flexible *CO configuration on PdCuZn NSs might explain the enhanced activity and selectivity after the simultaneous incorporation of Cu and Zn. According to previous reports, the sequence of *CO binding energy is *CO_L_ < *CO_B_ < *CO_T_.^21,22,58^ Therefore, *CO_L_ desorption more easily occurs because the binding affinity of *CO_L_ on Pd sites is weaker than that of *CO_B_ and *CO_T_ configuration. Too many *CO_T_ configurations on Pd sites lead to a strong binding affinity of *CO, which means poisoning of Pd sites, and finally harm the CO production. The quantitative analysis of the CO_L_/(CO_B_ + CO_T_) ratios is provided to evaluate the *CO binding strength. As shown in Figure 4e, PdCuZn NSs achieve the highest CO_L_/(CO_B_ + CO_T_) ratios compared with Pd, PdCu, and PdZn under all selected potentials. The increasing proportion of weak-binding *CO_L_ configuration demonstrates enhanced anti-CO-poisoning capability and makes subsequent *CO desorption easy to carry out on PdCuZn NSs, which eventually confers the impressive CO_2_-to-CO performance.
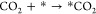
Step 1
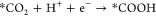
Step 2
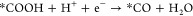
Step 3
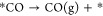
Step 4

Based on the above results, the specific reaction process over PdCuZn NSs for the CO_2_RR to the CO products is proposed in Figure 4f. First, CO_2_ molecules are absorbed and activated on the PdCuZn NS surface to form *CO_2_ (StepStep 1). Second, *CO_2_ is hydrogenated to generate *COOH intermediates (StepStep 2). Then, *CO is formed through the proton-coupled electron transfer (PCET) process (StepStep 3). The *CO on the PdCuZn surface is more likely to choose the *CO_L_ configurations after Cu and Zn doping, which is totally different from the dominant *CO_T_ configurations on Pd NSs. Such evolving tendencies on the *CO configurations highlight the significance of electronic modulation via the Cu and Zn coincorporation. Finally, CO gas molecules are generated due to facile *CO desorption from the active sites of the catalyst surface (StepStep 4).

Density functional theory (DFT) calculations were performed to gain in-depth insight into the enhanced catalytic mechanism toward CO_2_-to-CO conversion. First, the simulated structural models were constructed upon the fcc structure, which exposes (111) facets according to the TEM and XRD results. The modeling details are described in the Supporting Information, and the specific structural models of Pd, PdZn, PdCu, and PdCuZn are shown in Figure S24. Considering that there are three metal elements in PdCuZn, we first calculated the reaction pathways and free energies on Cu, Zn, and Pd sites of PdCuZn to determine the true active sites toward the CO_2_RR to CO process. For the CO_2_RR to CO process over both the Cu site and Pd site, the *CO_2_ absorption is the rate-determining step, which requires free energies of 0.38 and 0.29 eV on Cu and Pd sites, respectively. For the Zn site, the *CO_2_ hydrogenation to *COOH formation is the rate-determining step, which gives an increased energy barrier of 0.53 eV, thus limiting the CO_2_-to-CO conversion on the Zn site (Figure S25). Since the Pd sites of the PdCuZn surface exhibit the lowest energy barrier for the CO_2_RR to CO, the surface Pd sites of PdCuZn act as the most active sites for the CO_2_RR to CO.

**Figure 5:**
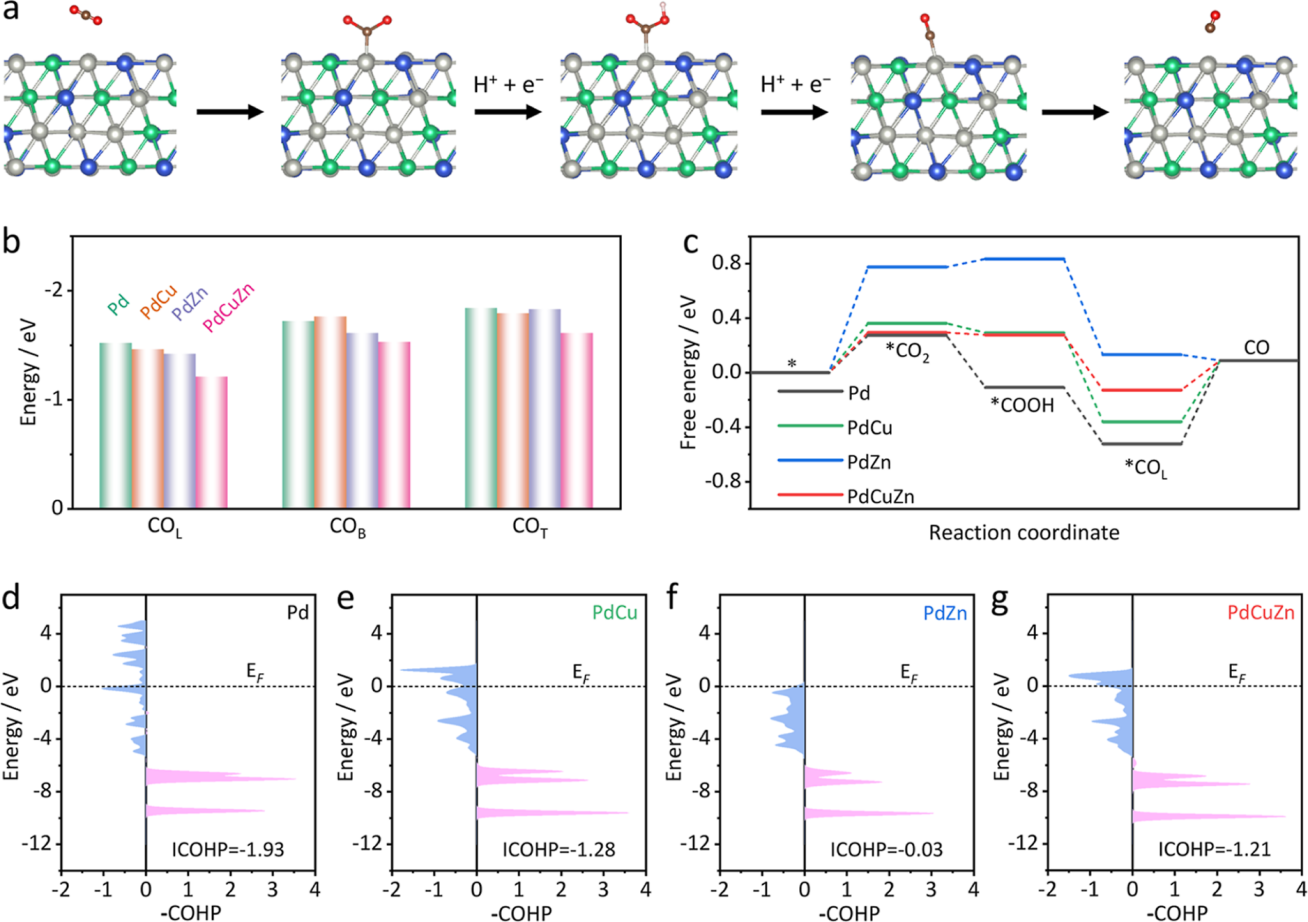
DFT simulations. (a) Reaction pathway of the CO_2_RR to CO over PdCuZn NSs (the adsorption of *CO intermediates displayed here is *CO_L_). (b) Adsorption energies of CO_L_, CO_B_, and CO_T_ on Pd, PdCu, PdZn, and PdCuZn. (c) Free energy diagrams of Pd, PdCu, PdZn, and PdCuZn via the *CO_L_ configuration. (d–g) COHP plots of different catalysts. Light purple represents the bonding state, and light blue represents the antibonding state.

The reaction pathway for the CO_2_RR to CO over PdCuZn is shown in Figure 5a, and the atomic configurations of adsorbates on the Pd sites of Pd, PdZn, and PdCu are shown in Figure S26, in which CO_2_ is converted to CO through the following elementary reaction steps including CO_2_ absorption, CO_2_ activation to *COOH, *CO formation, and *CO desorption. First, the *CO adsorption configurations (CO_L_, CO_B_, and CO_T_) on the surface of Pd, PdZn, PdCu, and PdCuZn were studied by DFT calculations. Specific structural models are shown in Figure S27. The corresponding adsorption energies of *CO_L_, *CO_B_, and *CO_T_ on Pd, PdCu, PdZn, and PdCuZn were summarized and are shown in Figure 5b. The *CO_L_ absorption configuration on the surface of Pd, PdZn, PdCu, and PdCuZn exhibits the lowest binding affinity, indicating that *CO_L_ is more easily desorbed compared with *CO_B_ and *CO_T_. Moreover, PdCuZn displays more positive adsorption energies for the three kinds of *CO adsorption than pure Pd, PdCu, and PdZn, indicating the weaker *CO absorption strength on the surface of PdCuZn than pure Pd, PdCu and PdZn.

The free energetics on Pd, PdCu, PdZn, and PdCuZn via the *CO_L_, *CO_B_, and *CO_T_ configurations for the CO_2_RR to CO were further calculated and are shown in Figures 5c and S28. The calculated Gibbs free energies of CO_2_ (ΔG_*CO_2__) on Pd sites over all catalysts are positive values (0.29, 0.36, 0.78, and 0.27 eV for PdCuZn, PdCu, PdZn, and Pd, respectively), indicating that the absorption of CO_2_ on active sites is the potential-determining step and PdCuZn could achieve CO_2_ absorption under lower potential compared with the bimetallic counterparts. The following *COOH formation (ΔG_*COOH_) reveals an exothermic step over Pd (−0.11 eV), PdCu (0.29 eV), and PdCuZn (0.27 eV), while it is endothermic on PdZn (0.84 eV). Then, the free energy pathways to *CO formation through PCET are thermodynamically downhill over all catalysts, implying that the *CO formation spontaneously happens over all catalysts. The subsequent *CO species are desorbed from the catalyst surface via the *CO_L_, *CO_B_, and *CO_T_ configurations. Obviously, the desorption energy barriers of *CO_L_ species are much lower than those of *CO_B_ and *CO_T_ species for all four catalysts, suggesting that the *CO_L_ configuration is more active than the other two configurations toward the formation of the gaseous CO product.

Specifically, PdZn shows the lowest energy barrier for *CO_L_, *CO_B_, and *CO_T_ desorption to release CO (g), while the larger energy barrier of *CO_2_ absorption (0.84 eV) limits its CO_2_RR to CO production. The desorption of *CO_L_, *CO_B_, and *CO_T_ species to form CO (g) on Pd, PdCu, and PdCuZn is found to be thermodynamically uphill. Compared with PdCu and Pd, PdCuZn exhibits the lowest energy barrier for *CO_L_, *CO_B_, and *CO_T_ desorption to produce CO (g). These results also indicate that no matter what *CO configuration is adopted during the CO_2_RR to CO, PdCuZn always exhibits the lowest energy barrier for the CO_2_-to-CO conversion when compared with other Pd-based counterparts, implying that the bimetallic doping strategy is effective in facilitating the CO_2_RR to CO production.

The projected density of states (PDOS) was then calculated to investigate the adsorption and binding behaviors of intermediates upon the electronic structures. As is shown in Figure S29, the d-band centers of PdCuZn (3.18 eV), PdCu (2.12 eV), and PdZn (2.99 eV) display a downward shift relative to the pure Pd (1.80 eV), in good conformity with the VB-XPS analysis. And the PDOSs of each element in PdCuZn reveal their electronic contributions and confirm the existence of strong electronic coupling (Figure S30a). In addition, Bader charge analysis of PdCuZn shows that the charge state of Pd is positive, while the charge states of Cu and Zn are negative (Figure S30b), also suggesting strong electronic coupling between multicomponent elements.

In order to further study the bonding properties between Pd sites and *CO species, we calculated the crystal orbital Hamilton population (COHP) between Pd active sites and *CO.^59,60^ As shown in Figure 5d–g, the antibonding orbitals below the Fermi level in PdCu, PdZn, and PdCuZn are more occupied than pure Pd, signifying the weak bonding strengths between Pd sites and *CO in PdCu, PdZn, and PdCuZn. The orbital overlaps and bonding strengths between Pd and *CO were further quantitatively analyzed by integrated COHP (ICOHP), which is computed by integrating the COHP up to the Fermi level. The ICOHP values of Pd, PdCu, PdZn, and PdCuZn are −1.93, −1.28, −0.03, and −1.21 eV, respectively. The increased ICOHP values further demonstrate the weaker bonding strengths between Pd sites and *CO in PdCu, PdZn, and PdCuZn compared with pure Pd. Furthermore, CO stripping measurements were carried out to experimentally study the absorption of CO on as-prepared catalysts. As shown in Figure S31, the PdCuZn NSs exhibit an obvious shift of the CO oxidation peak (∼27 mV shift to lower potentials) and a dramatical increase of peak intensity, confirming the weaker CO adsorption and higher CO resistance on PdCuZn NSs than that of pure Pd NSs. The combined results of DFT simulations and CO stripping experiments emphasize that the interaction between Pd and adsorbed CO is prominently diminished after the codoping of Cu and Zn into the Pd lattice, thereby expediting the CO desorption and facilitating the conversion of CO_2_RR to CO.

## 3 Conclusions

In summary, ultrathin Pd-alloy electrocatalysts with alleviated *CO binding affinity have been subtly designed and synthesized for efficient CO_2_-to-CO conversion. Simultaneous codoping of Cu and Zn into the Pd atomic layer generates more effective outcomes for fine control of the catalyst’s electronic structure when compared with the bimetallic PdCu, PdZn, and pure Pd NSs. The as-resulted PdCuZn NSs with few atomic layers exhibit highly selective CO production under an extended potential window, achieving an optimal FE_CO_ of 96% at −0.35 V (vs RHE). The PdCuZn electrode also maintains a CO production of ∼95% FE_CO_ during 100 h electrolysis, revealing its potential for practical CO_2_RR applications. In situ electrochemical ATR-FTIR spectroscopy measurements manifest a fast *CO desorption process over PdCuZn NSs due to the selected adoption of a weakly bound *CO_L_ configuration on the NS surface, leading to the enhanced anti-CO-poisoning capability. DFT calculations confirm that coincorporation of Cu and Zn into the Pd lattice produces a more reasonable electronic structure and lower d-band center of active sites, which effectively tailors the adsorption configuration of intermediates and decreases the energy barrier of rate-determining *CO desorption, thereby accelerating the CO_2_-to-CO conversion. This study highlights a principle for designing and synthesizing Pd-based alloy electrocatalysts, in which the bimetallic codoping tactic enables precise modification of adsorption configurations to meet the appetite of selective CO_2_RR applications.

## Supporting Information Available

Detailed experimental and calculation information, additional material characterizations, electrochemical data, and theoretical simulation data (PDF)
