# Supplementary material for: Steering the Absorption Configuration of Intermediates over Pd-Based Electrocatalysts toward Efficient and Stable CO2 Reduction
Source: J Am Chem Soc. 2025 Jan 24;147(5):4219–29. doi: 10.1021/jacs.4c14253 (PMC11803748; doi:10.1021/jacs.4c14253)
Supplement: Supplementary file 1 — ja4c14253_si_001.pdf [file ja4c14253_si_001.pdf]

Supporting Information

Steering the Absorption Configuration of Intermediates over Pd-based Electrocatalysts toward Efficient and Stable CO_2 Reduction

Shuting Wei[1,3], Yanchao Xu[1], Tao Song[2], Hao Dai[2], Fan Li[2], Xin Gao[3], Yanjie Zhai[3], Shanhe Gong[3], Rui Li[4], Xiao Zhang[3,5,*], Kangcheung Chan [1,5,*]

[1]Department of Industrial and Systems Engineering, The Hong Kong Polytechnic University, Kowloon 999077, Hong Kong, China

[2]Department of Chemistry, Southern University of Science and Technology, Shenzhen, 518055, China

[3]Department of Mechanical Engineering, The Hong Kong Polytechnic University, Kowloon 999077, Hong Kong, China

[4]Institute of Clean Energy, Yangtze River Delta Research Institute, Northwestern Polytechnical University, Xi'an 710072, China

[5]Research Institute for Advanced Manufacturing, The Hong Kong Polytechnic University, Kowloon 999077, Hong Kong, China

*Corresponding authors: E-mail: xiao1.zhang@polyu.edu.hk; kc.chan@polyu.edu.hk

1. Experimental sections

1.1 Chemicals

The palladium(II) acetylacetonate (≥99.9%), copper(II) acetylacetonate (≥99.9%), zinc(II) acetylacetonate was purchased from Sigma-Aldrich. The potassium hydroxide (electronic grade, 99.99% metals basis) and potassium bicarbonate (≥99.99% metals basis) were purchased from Macklin Biochemical Technology Co., LTD. Ethanol, N,N-dimethylformamide, ethylene glycol and diethylenetriamine were purchased from Sinopharm Chemical Regent. 1H,1H,2H,2H-perfluorooctyltrichlorosilane was purchased from Energy Chemical. All reagents are directly used without further purification treatment. The deionized water (18.2 MΩ cm) was used throughout overall experiments.

1.2 Preparation of PdCuZn nanosheets

The synthesis of Pd nanosheets as described in the previous report with minor modifications.[1] 2.5 mg of palladium acetylacetonate and 0.8 g of potassium hydroxide were dispersed into the mixed solvent containing 6.0 mL of N,N-dimethylformamide, 4.0 mL of ethylene glycol and 5.0 mL of diethylenetriamine. Subsequently, the mixed solvent was transferred to Teflon-lined autoclave and sealed, then kept at 200 ℃ for 5 h. The product was collected, washed with ethanol and water and finally dried in a vacuum oven at 60 ℃ for 12 h.

The synthesis of PdCuZn nanosheets: 2.5 mg of palladium acetylacetonate, 1.0 mg of copper acetylacetonate, 1.0 mg of zinc acetylacetonate and 0.8 g of potassium hydroxide were dispersed into the mixed solvent containing 6.0 mL of N,N-dimethylformamide, 4.0 mL of ethylene glycol and 5.0 mL of diethylenetriamine. Subsequently, the mixed solvent was transferred to Teflon-lined autoclave and sealed, then kept at 200 ℃ for 5 h. The products were collected, washed with ethanol and water and finally dried overnight at 60 ℃ in a vacuum oven. PdCu and PdZn nanosheets were synthesized according to the above method by feeding corresponding metal salts.

1.3 Characterization

Transmission electron microscope (TEM) images, high resolution TEM (HR-TEM), corresponding energy dispersive X-ray spectroscope (EDS) analysis were performed by applying a Talos F200X G2 (Thermo Fisher, USA). Scanning electron microscopy (SEM) image was obtained on a Field emission scanning electron microscope (Tescan MAIA3, Europe). XRD pattern were conducted on an X-ray diffractometer (Rigaku Smartlab, Japan) equipped with a Cu Kα emitting source. The thickness of nanosheet was carried out on the Asylum Research Cypher system AFM (Oxford instruments, USA). ^{1}H-NMR spectroscopy was

performed on a Bruker 400 MHz spectrometer. The metal element contents were tested by inductively coupled plasma optical emission spectrometer (ICP-OES) (Agilent 720ES, USA). X-ray absorption spectroscopy (XAS) of the Pd K-edge, Cu K-edge and Zn K-edge were tested in transmission mode at the BL-17C and 01C1 beamline station of National Synchrotron Radiation Research Center (NSRRC), Hsinchu, Taiwan. X-ray photoelectron spectroscopy (XPS) analysis was executed on a PHI 5000 VersaProbe III apparatus (ULVAC PHI, Japan) with a monochromatic Al-Kα radiation source. According to the XPS valence band spectra, the d-band center of the samples was evaluated in the range of −1 to 8 eV by applying the following formula:

$$d\text{-}band\ center = \frac{\int N(\varepsilon)\varepsilon d\varepsilon}{\int N(\varepsilon) d\varepsilon} \quad (1)$$

where $N(\varepsilon)$ is the density of states of the occupied d-states, ε is the energy of state.

1.4 Electrode preparation

10.0 mg of as-prepared catalyst was dispersed into 2.0 mL of ethanol containing 20.0 μL of Nafion solution (5%), followed by ultrasonication for one hour. The homogeneous solution was sprayed on gas diffusion layer (GDL) electrode (YLS-30T). The loading amount of catalyst is 1.0 mg cm^{-2} based on the weight of GDL before and after spraying.

1.5 Electrochemical Measurement

The electrochemical performance was tested with a CHI760e electrochemical workstation. All electrochemical measurements were carried out under ambient temperature and pressure. A three-compartment flow cell composed of a cathode chamber, anode chamber and gas chamber were utilized for CO_2RR. The catalyst/GDL, IrO_2/Ti electrode and Ag/AgCl electrode were used as the working electrode, counter electrode and reference electrode, respectively. 20 sccm of high purity CO_2 gas is fed to a gas chamber and transported to the catalyst layer through the back side of GDL. An anion-exchange membrane (FAB-PK-130) was used to separate the anode electrode and cathode electrode. 1.0 M KOH was circulated as electrolyte in cathode chamber and anode chamber through the peristaltic pumps. All the applied potentials (E) mentioned were tested against the Ag/AgCl electrode and converted to reversible hydrogen electrode (RHE) on the basis of this equation with iR compensation:

$$E_{RHE} = E_{Ag/AgCl} + 0.0591 \times pH + 0.197 - 0.85 \times iR \quad (2)$$

where E_{RHE} is the corrected potential, $E_{Ag/AgCl}$ is the applied potential, i is the total current, 0.85 is an applied correction factor, R is the resistance between the reference electrode and the working electrode measured via electrochemical impedance spectroscopy, which was

conducted with frequency range from 100 kHz to 0.1 Hz and an amplitude of 5 mV at open circuit voltage.

The electrochemically active surface area (ECSA) was recalculated based on the charges of Pd oxide reduction peak from recorded cyclic voltammetry (CV). The CV tests were performed in Ar-saturated 1.0 M KOH solution between 0.05~1.25 V vs. RHE at 50 mV s^{-1}, and the ECSAs were determined by the integrated charge of oxide reduction according to the following equation:

$$ECSA = \frac{Q}{\theta} \quad (3)$$

where Q (mC) is the charge for the Pd oxide reduction. θ (0.42 mC cm^{-2}) is the electrical charge related to full monolayer adsorption of O/OH on Pd.

The long-term electrolysis in a three-compartment flow cell was conducted at ambient temperature and pressure in 1.0 M KOH. To increase the hydrophobicity of GDL and to improve the stability of GDE, 20 μL of 1H,1H,2H,2H-perfluorooctyltrichlorosilane (5 wt% in toluene) was modified on the back of GDL before the measurement. The electrolyte was also constantly refreshed to avoid possible salt accumulation during testing. And CO gas was collected and analyzed every 5 h by on-line gas chromatography.

Stability test in MEA flow cell: The MEA electrolyser is made up of a cathode electrode (catalyst-coated GDL), anion exchange membrane (Sustainion X37-50 Grade RT Membrane) and anode electrode (nickel foam). The cathode electrode and anode electrode are separated by anion exchange membrane and assembled in the MEA electrolyzer. The titanium plates were used to make anode and cathode plates, and the geometric active areas of middle flow channel is 1 cm^2. The CO_2 gas with a flow rate of 20 sccm was transported to the cathode electrode along the cathode flow channel, while KOH electrolyte was circulated on the anode flow channel with a flow rate of 100 rpm using peristaltic pumps. The long-time durability for CO_2RR was performed at −200 mA cm^{-2}. The electrolyte was also constantly refreshed during the testing process, while CO gas was monitored every 5 h by on-line gas chromatography.

1.6 In situ ATR-FTIR measurements

In situ ATR-FTIR spectra were recorded on a Bruker V80 infrared spectrometer equipped with liquid nitrogen-cooled LN-MCT Mid detector. The spectral tests were performed in a home-made IR spectroelectrochemical H-type reaction cell. The catalyst ink was dropped on the hemisphere silicon prism coated with gold film as the working electrode. An Ag/AgCl electrode and a Pt foil were used as reference electrode and counter electrode, respectively. CO_2-saturated 0.2 M $KHCO_3$ was used as the electrolyte and CO_2 was continuously purged during the testing process. The ATR-FTIR spectra data were collected from 4000 to 1000 cm^{-1} with a spectral

resolution of 4 cm^{-1} during the linear sweep voltammetry test between 0.1 V ~ −0.8 V vs. RHE. The infrared spectrum at open circuit potential was recorded and acted as a reference spectrum. In situ ATR-FTIR spectroscopy data of PdCuZn NSs under Ar atmosphere was conducted under the same conditions as described above, except that Ar was used instead of CO_2.

1.7 Products analysis

The gas products were detected gas chromatography (FULI GC9790PLUS) equipped with Hayesep A, Porapak N and molecular sieve 5A columns. High purity Argon (99.99%) was used as the carrier. Hydrogen was quantitatively analysed by a thermal conductivity detector (TCD), while carbon monoxide was quantitatively detected by a flame ionization detector (FID). The detector temperature was held at 150 °C. Calibration curves for H_2 and CO were obtained by injecting known quantities of standard gases. The concentration of gaseous products was quantitatively calculated using the integrated area of reduction products.

The Faradaic efficiency (FE) of the gas products were calculated according to the following equation:

$$FE(\%) = \frac{Q_{co}}{Q_{tot}} \times 100\% = \frac{\left(\frac{v}{60\ s/min}\right)\times\left(\frac{y}{24000\ cm^3/mol}\right)\times N\times F}{j} \times 100\% \quad (4)$$

Where, v is the CO_2 gas flow rate (sccm), y is the concentration of product detected by GC, N is the number of transfer electron, F is the Faraday constant (96485 C mol^{-1}), j is the testing current (A).

The liquid products were detected by ^{1}H-NMR spectroscopy on Bruker 400M Nuclear magnetic resonance (NMR) spectrometer with dimethyl sulfoxide (DMSO) as the internal standard, in which 0.40 mL of the electrolyte was mixed with 0.05 mL of deuterated water and 0.05 mL, 80 ppm of DMSO.

2. Theoretical calculation

All the calculations were performed in the framework of the density functional theory with the projector augmented plane-wave method, as implemented in the Vienna ab initio simulation package.[2] The generalized gradient approximation proposed by Perdew, Burke and Ernzerhof were selected for the exchange-correlation potential.[3] The electron-ion interaction was described with Pd (4*d*, 5*s*), Cu (3*d*, 4*s*) and Zn (3*d*, 4*s*) as valence electronic configurations. The cut-off energy for plane wave was set to 500 eV. The energy criterion was set to 10^{-5} eV in iterative solution of the Kohn-Sham equation. All crystal structure models for calculation were constructed by adopting the *fcc* crystal structure based on the experimental stoichiometry and the (111) facets were modeled according to the experimental results. The vacuum layer of 15 Å was added perpendicular to the sheet to avoid artificial interaction between periodic

images. The Brillouin zone integration was performed using a 3×3×1 k-mesh. All the structures were relaxed until the residual forces on the atoms have declined to less than 0.01 eV $Å^{-1}$.

The adsorption energies (E_{ads}) of CO on Pd-based samples were calculated according to the following equation (5):

$$E_{\mathrm{ads}} = E_{\mathrm{sub+CO}} - E_{\mathrm{CO}} - E_{\mathrm{sub}} \quad (5)$$

where E_{sub+CO} is the total energy of the system, E_{*CO} is the energy of CO molecule, and E_{sub} is the energy of the clean substrate.

The free energies for adsorbed species at 298 K and 1 atm were calculated by the following equation (6):

$$G = E_{DFT} + ZPE + H - TS \quad (6)$$

where E_{DFT} is the DFT total energy, ZPE is the zero-point energy, H is the integrated heat capacity, T is the reaction temperature and S is the entropy. The H and S were obtained from the references.[4,5] The gas phase corrections were obtained from the references.[6]

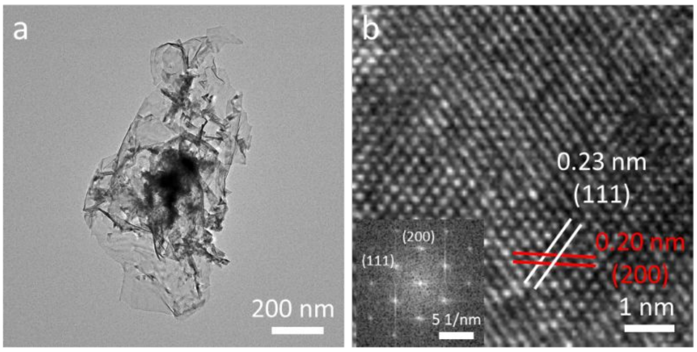

Figure S1. a) TEM and b) HRTEM image of Pd NSs.

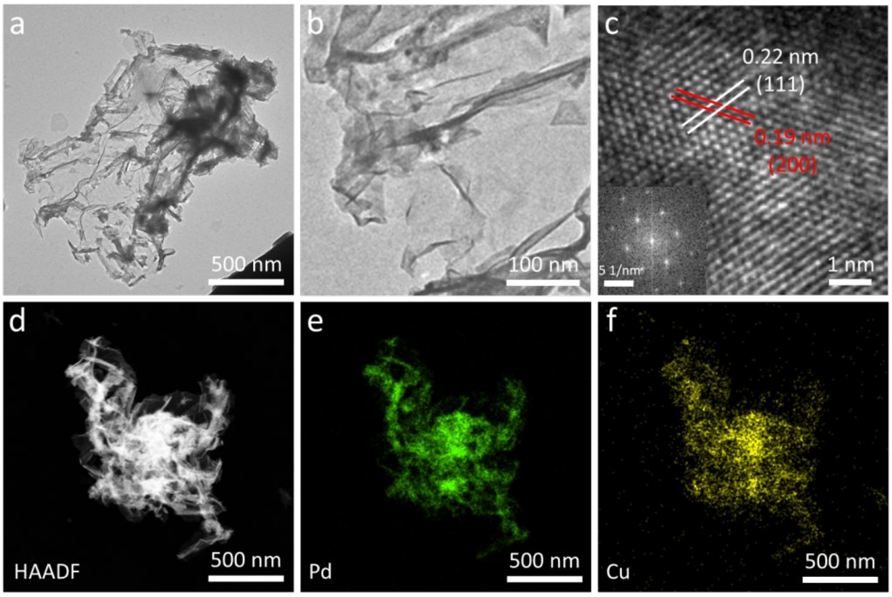

Figure S2. a-b) TEM images, c) HRTEM image, d) HAADF-STEM image and e-f) elemental mapping patterns of PdCu NSs.

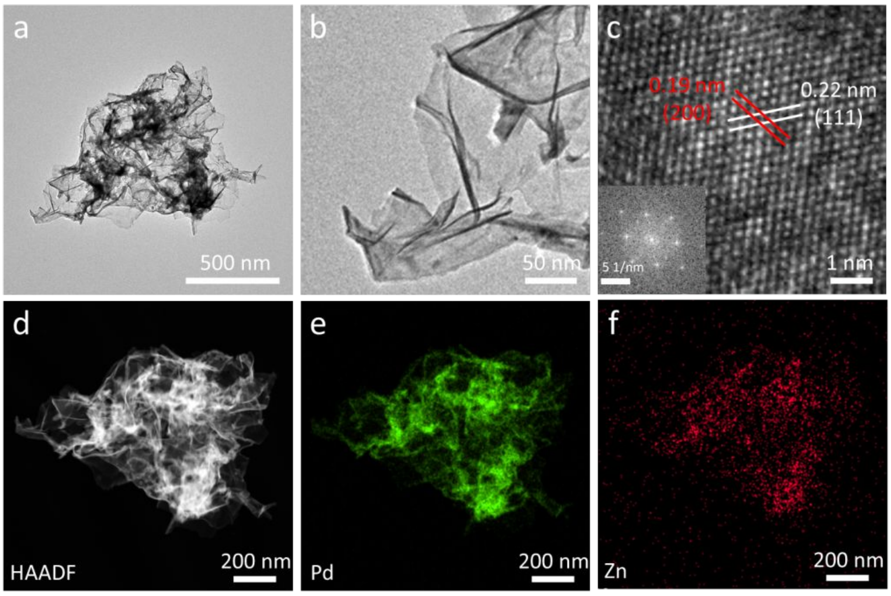

Figure S3. a-b) TEM images, c) HRTEM image, d) HAADF-STEM image and e-f) elemental mapping patterns of PdZn NSs.

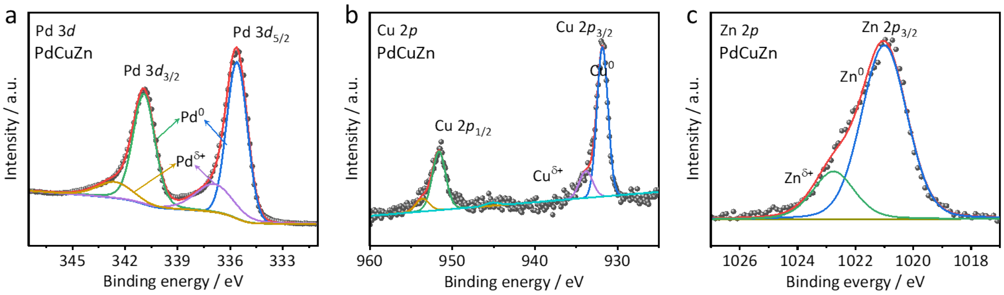

Figure S4. XPS spectra of PdCuZn NSs. a) Pd 3*d*, b) Cu 2*p* and c) Zn $2p_{3/2}$.

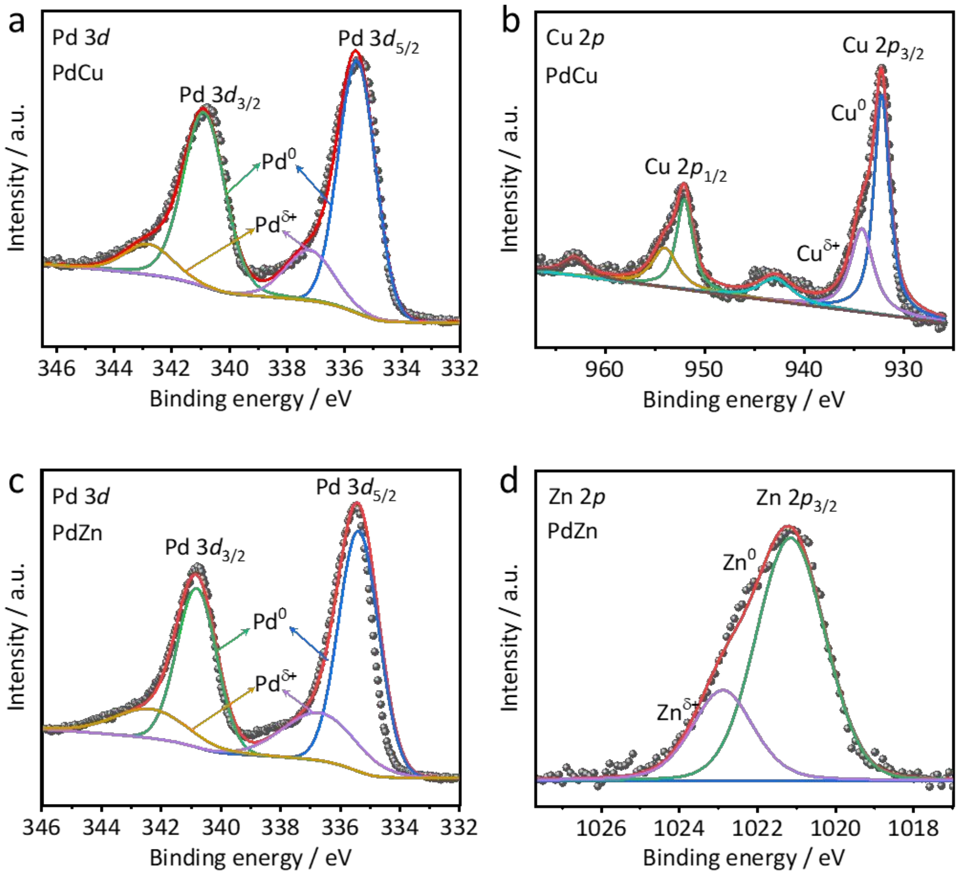

Figure S5. a) Pd 3*d* and b) Cu 2*p* XPS spectra of PdCu NSs, c) Pd 3*d* and d) Zn $2p_{3/2}$ XPS spectra of PdZn NSs.

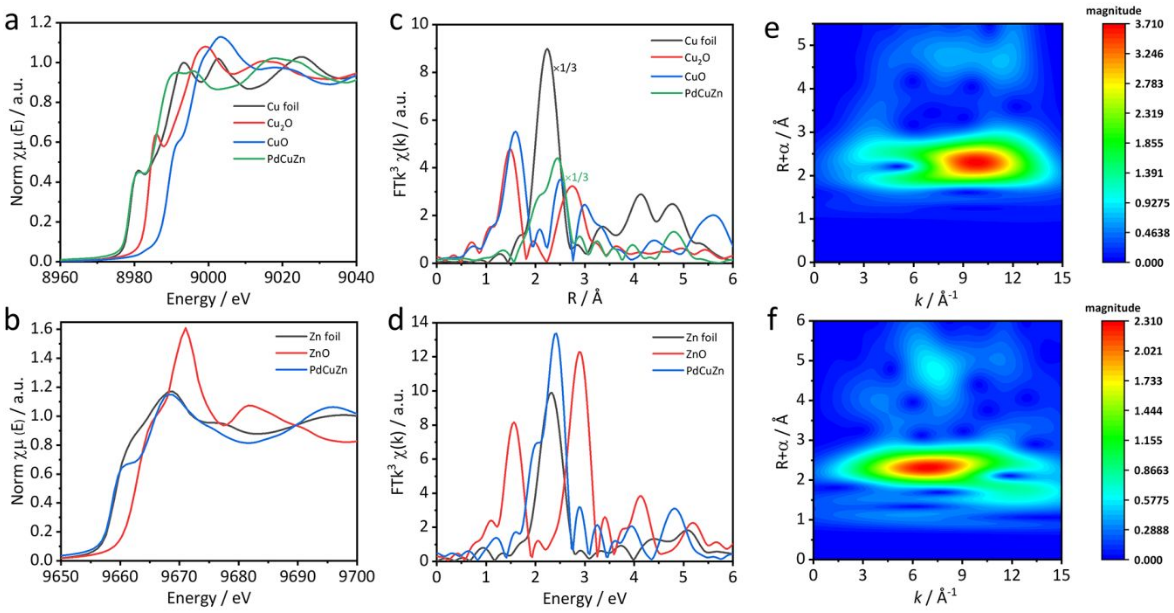

Figure S6. a) XANES spectra, c) FT-EXAFS spectra of the Cu K-edge region and e) WT-EXAFS pattern at Cu K-edge for PdCuZn NSs. b) XANES spectra, d) FT-EXAFS spectra of the Zn K-edge region and f) WT-EXAFS pattern at Zn K-edge for PdCuZn NSs.

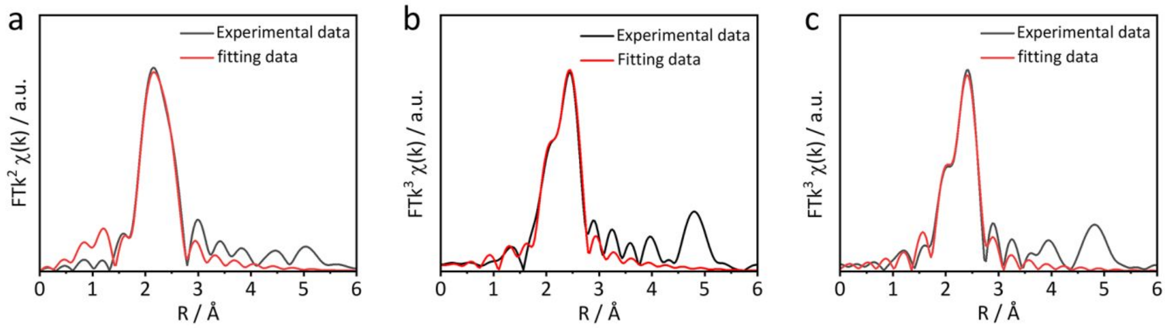

Figure S7. FT-EXAFS fitting results of a) Pd K-edge, b) Cu K-edge, and c) Zn K-edge for PdCuZn.

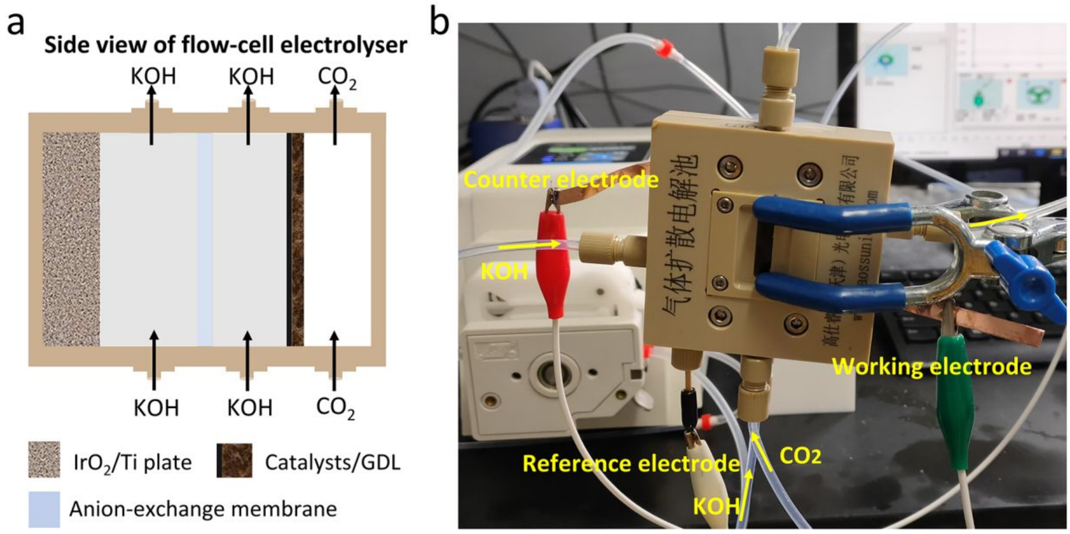

Figure S8. a) Schematic diagram and b) photograph of the three-compartment flow cell.

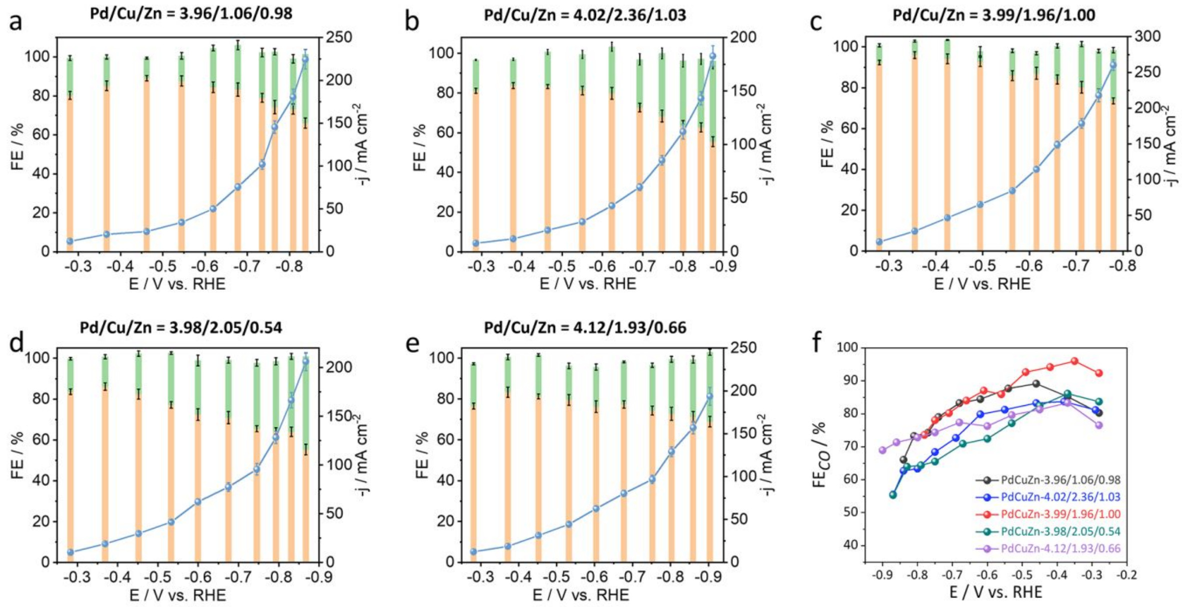

Figure S9. a-e) Electrochemical CO_2RR performance of Pd-based catalysts with different compositions. (Light orange: CO, Light green: H_2). f) The plots of FE_{CO} for PdCuZn catalysts with different compositions at various potentials.

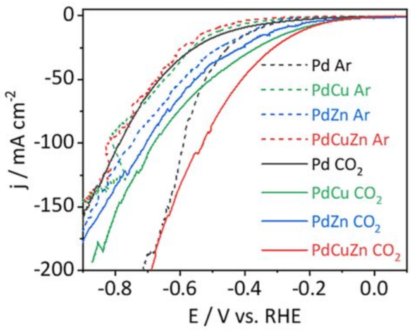

Figure S10. The polarization curves of as-prepared catalysts under different gas atmospheres.

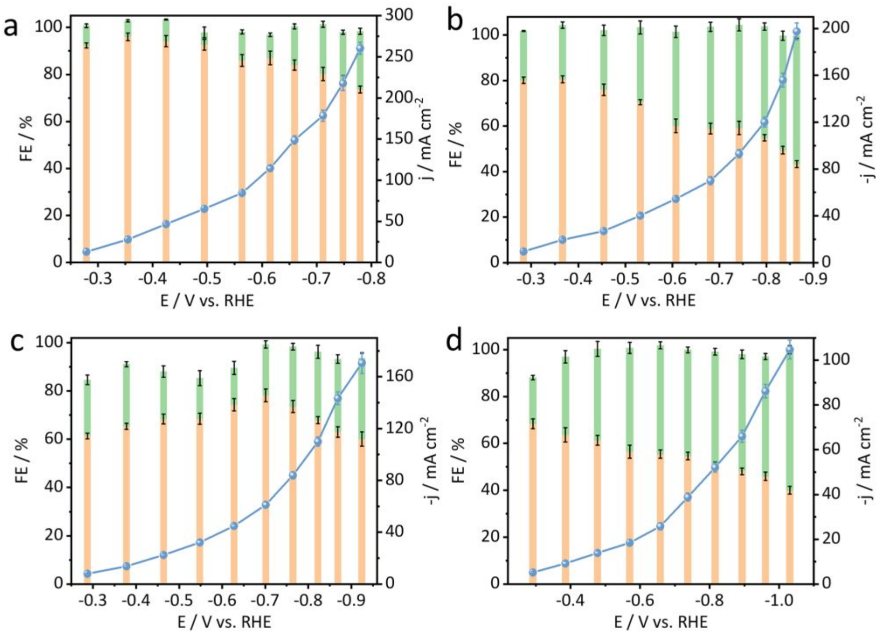

Figure S11. The FEs of CO_2RR products and corresponding current densities of a) PdCuZn, b) PdCu, c) PdZn and d) Pd NSs at different potentials (Light orange: CO, Light green: H_2).

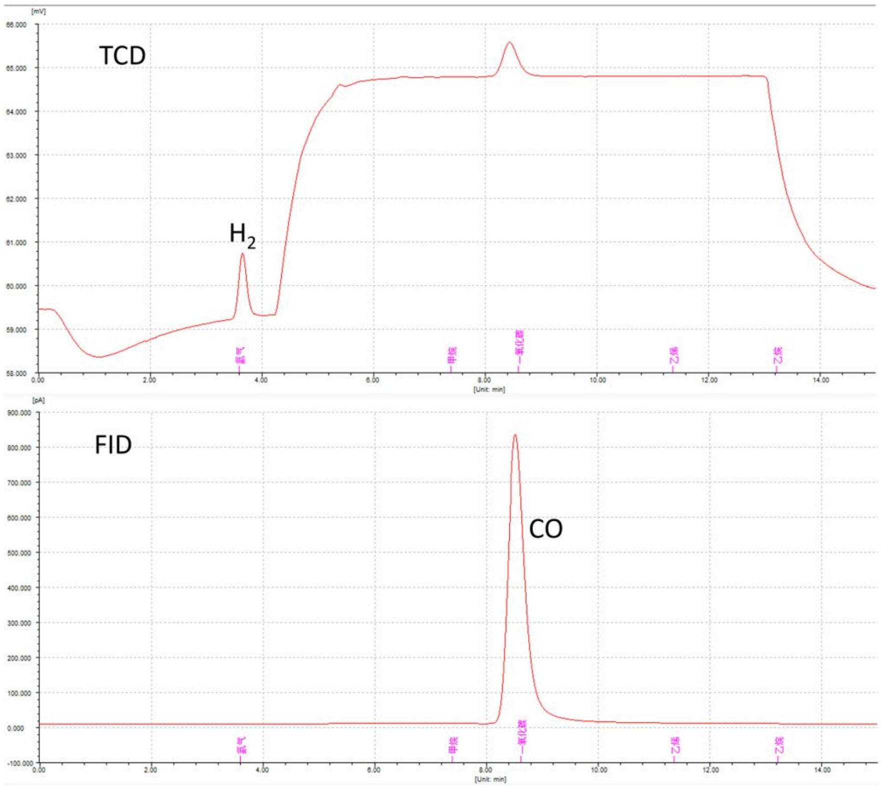

Figure S12. TCD and FID data of PdCuZn NSs for CO_2RR at −0.35 V.

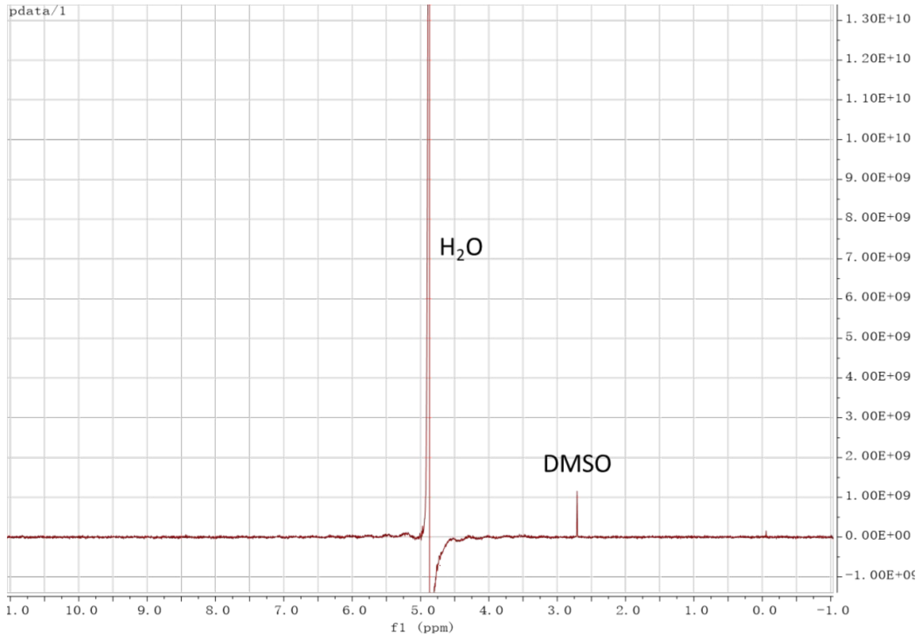

Figure S13. ^{1}H-NMR spectroscopy of the electrolyte solution after CO_2RR.

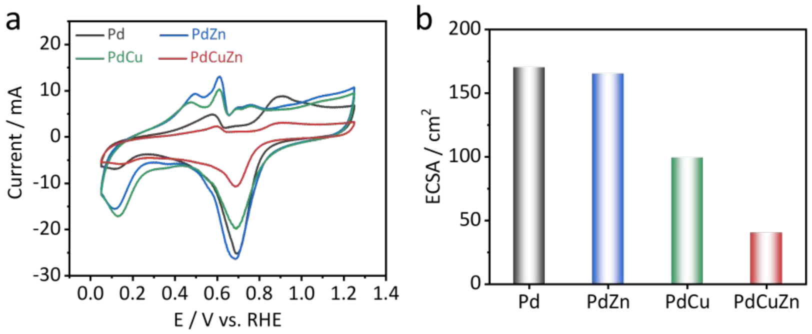

Figure R14. a) The CV curves of Pd-based catalysts recorded in Ar-saturated 1.0 M KOH solution. Scan rate: 50 mV s^{-1}. b) Summarized ECSA values.

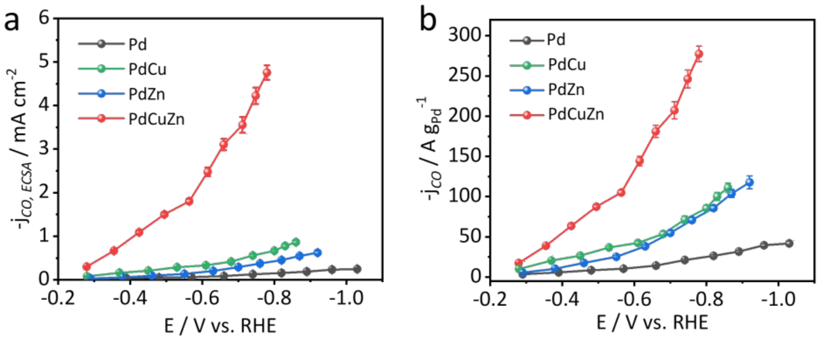

Figure S15. a) ECSA and b) mass-normalized activity of different catalysts.

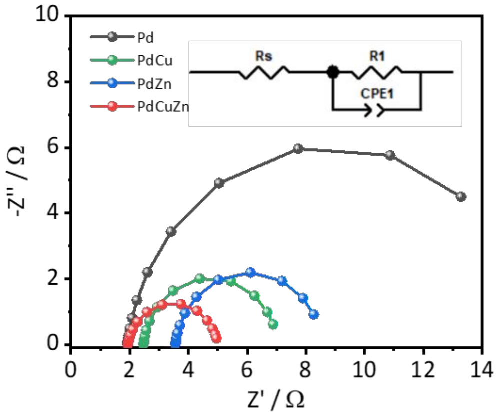

Figure S16. Nyquist plot of four catalysts (inset shows the equivalent resistance model used to fit the Nyquist curves).

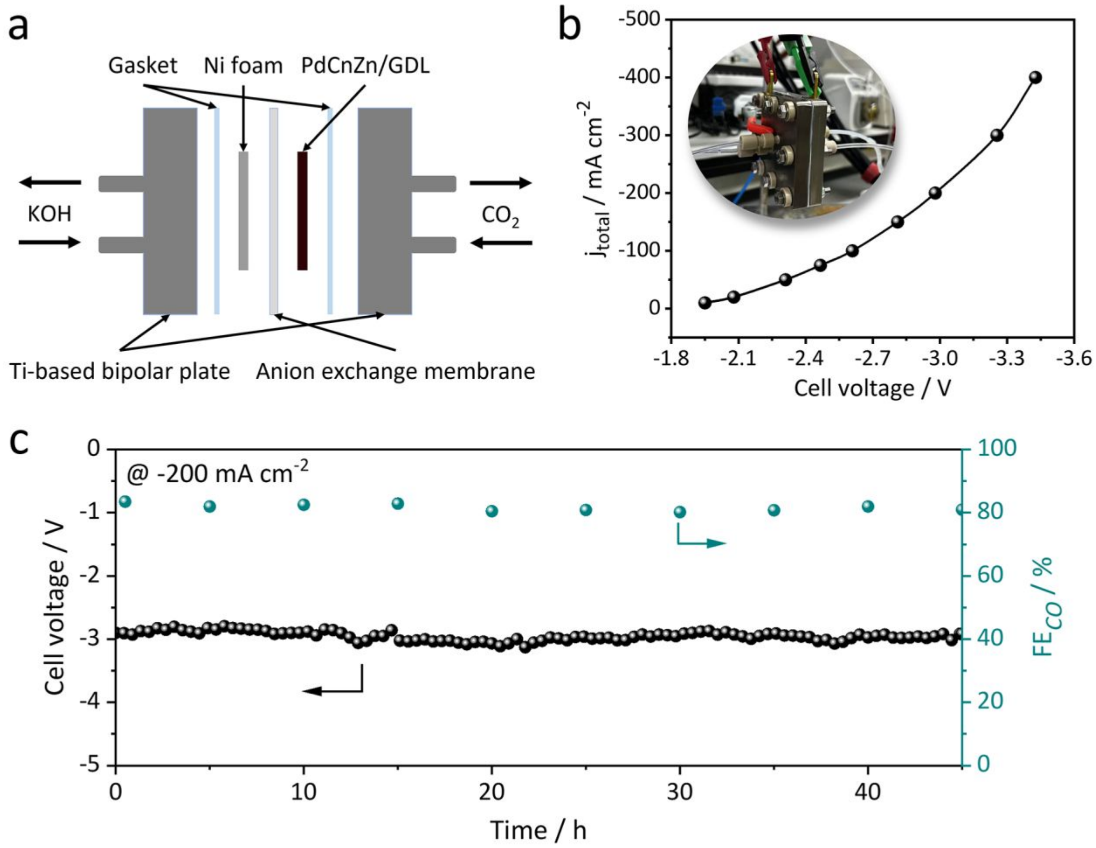

Figure S17. a) Schematic illustration of the MEA system. b) j_{total}-V plots with no *i*R compensation and c) stability test at −200 mA cm^{-2} in MEA flow cell.

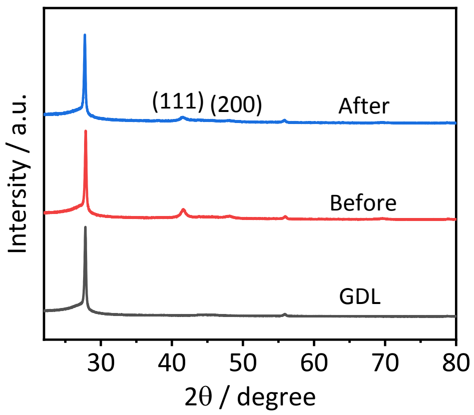

Figure S18. XRD pattern of PdCuZn before and after electrolysis.

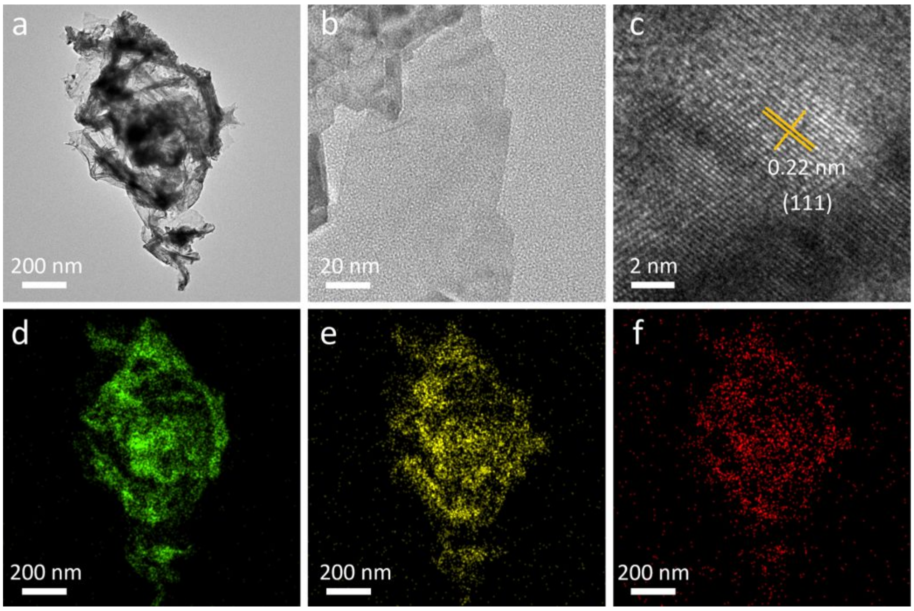

Figure S19. TEM characterization of PdCuZn after electrolysis.

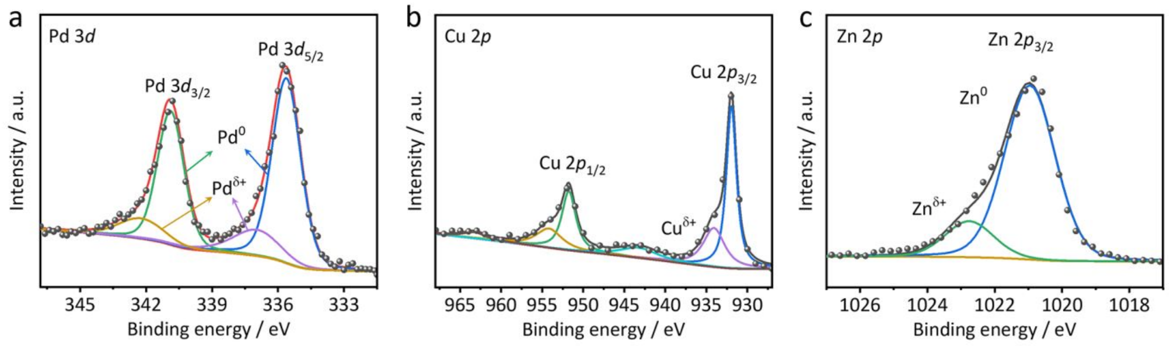

Figure S20. XPS spectra of PdCuZn after electrolysis. a) Pd 3*d*, b) Cu 2*p* and c) Zn $2p_{3/2}$.

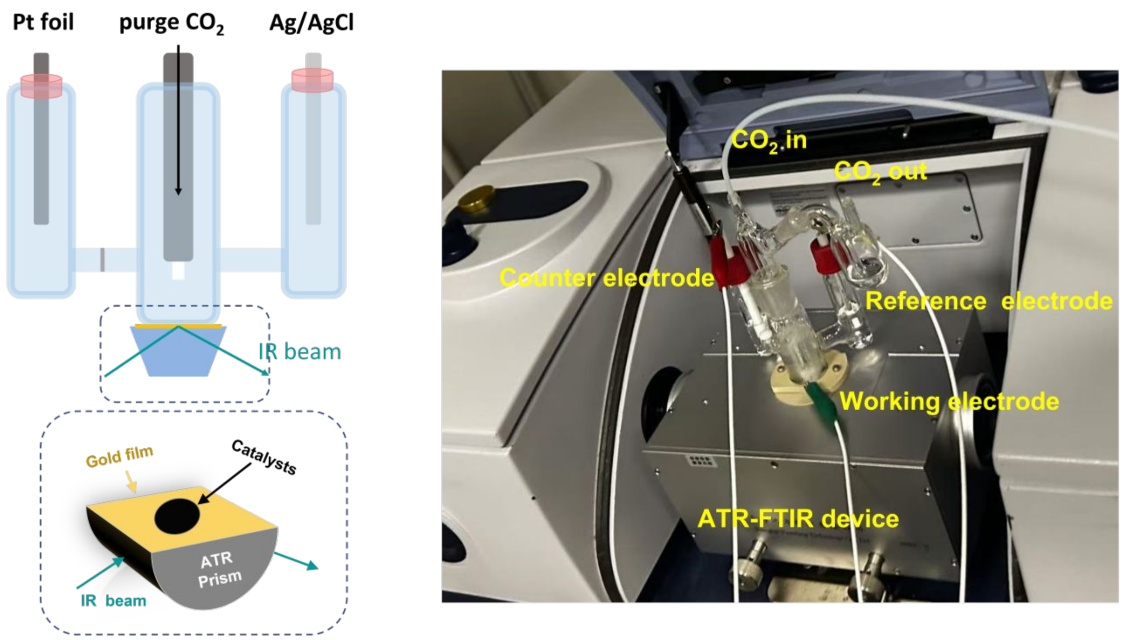

Figure S21. a) Schematic diagram of the working module and b) photograph of in situ ATR-FTIR setup.

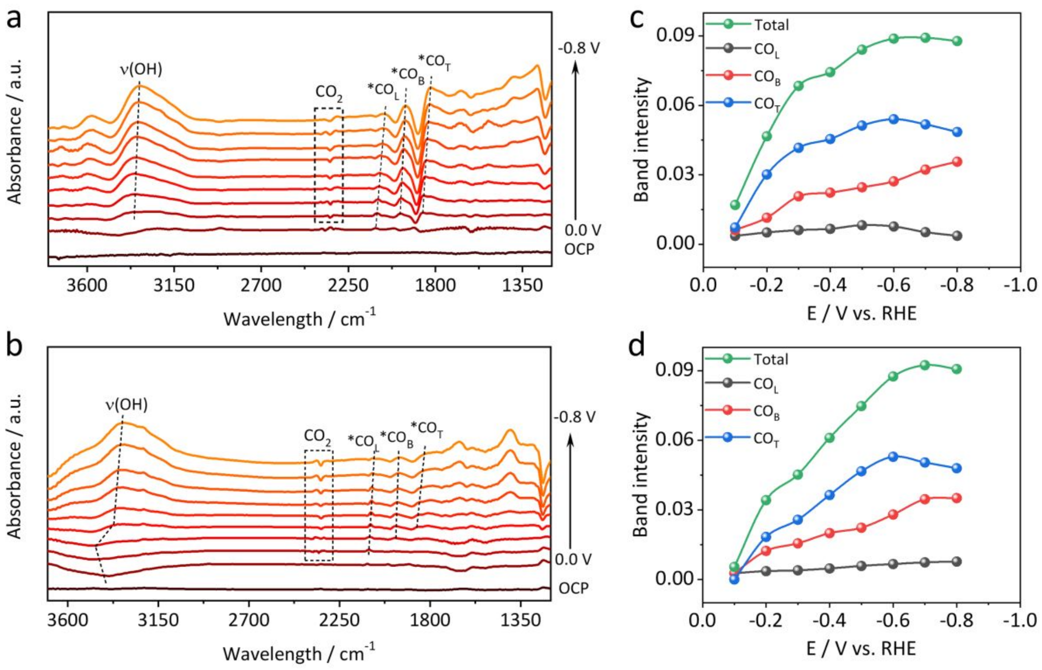

Figure S22. In situ ATR-FTIR spectroscopy of a) PdCu and b) PdZn. Potential-dependent *CO bands intensity of c) PdCu and d) PdZn.

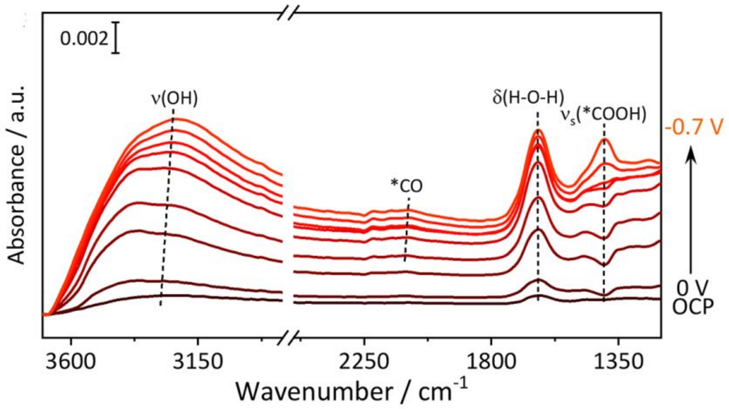

Figure S23. In situ ATR-FTIR spectra of PdCuZn NSs under Ar gas atmosphere.

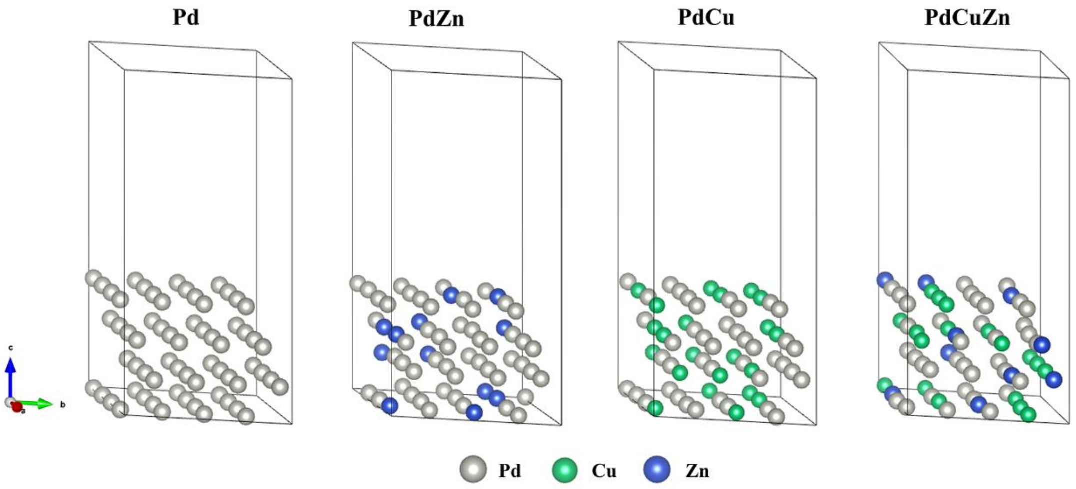

Figure S24. The structure modes of Pd, PdZn, PdCu and PdCuZn.

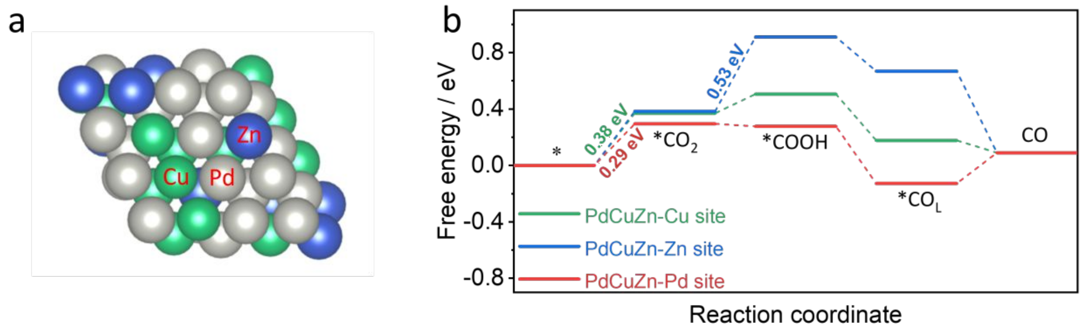

Figure S25. a) The structure mode of PdCuZn. b) Free energy diagrams on Pd, Cu, Zn site of PdCuZn.

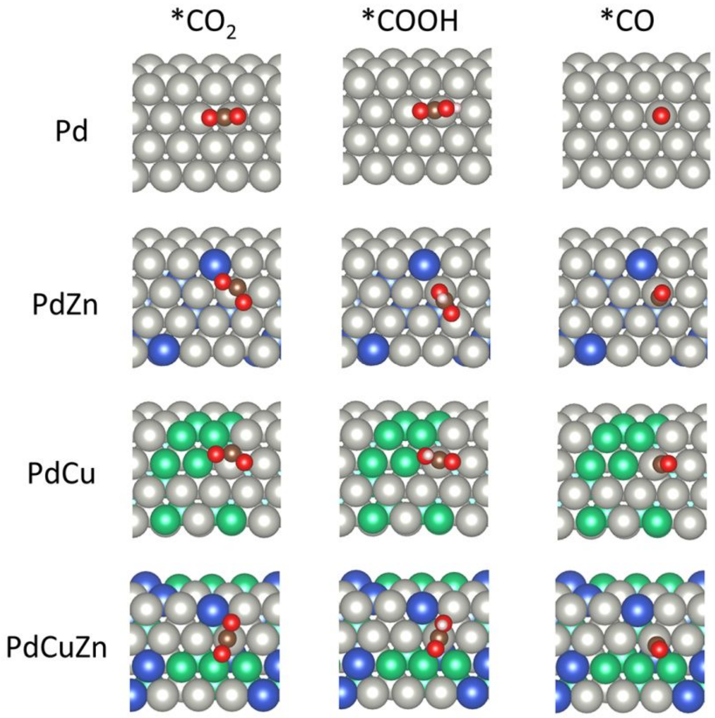

Figure S26. The atomic configurations of intermediates for CO_2RR on Pd, PdZn, PdCu and PdCuZn.

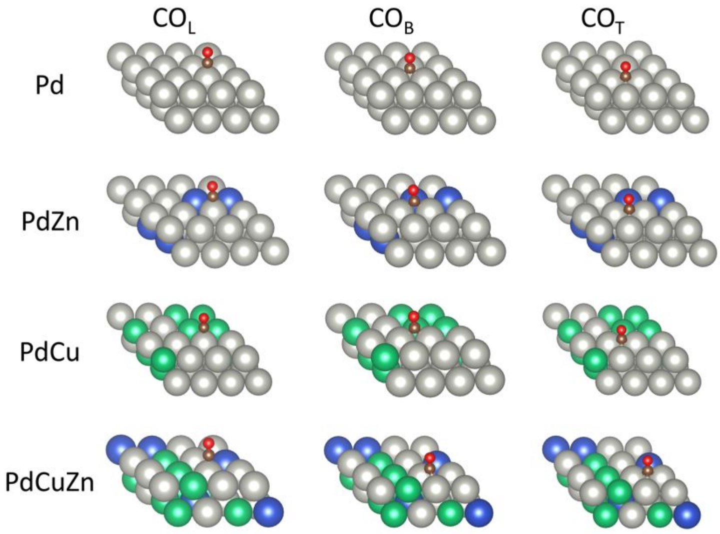

Figure S27. CO adsorption configurations (CO$_L$, CO$_B$, CO$_T$) on Pd, PdCu, PdZn and PdCuZn.

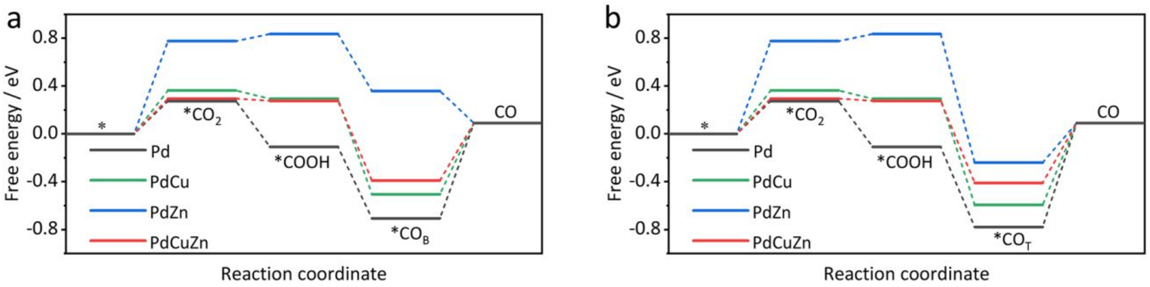

Figure S28. Free energy diagrams of Pd, PdCu, PdZn and PdCuZn for CO_2RR to CO via a) $*CO_B$ and b) $*CO_T$ configurations.

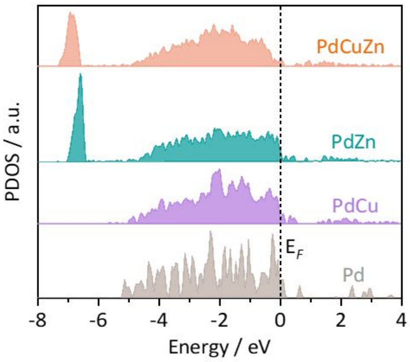

Figure S29. PDOSs of Pd, PdCu, PdZn and PdCuZn.

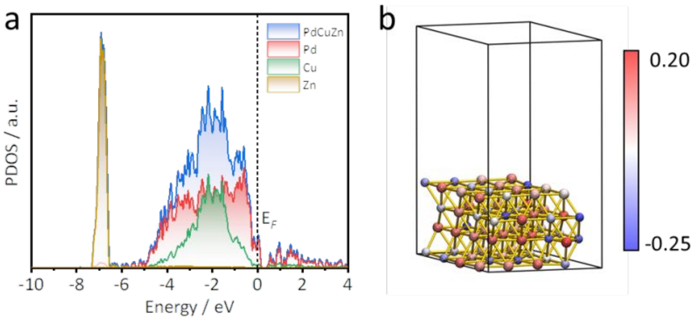

Figure S30. a) PDOS of PdCuZn and b) Bader charge analysis on PdCuZn structure catalysts. The positive value represents electron loss and negative value represents electron acquisition.

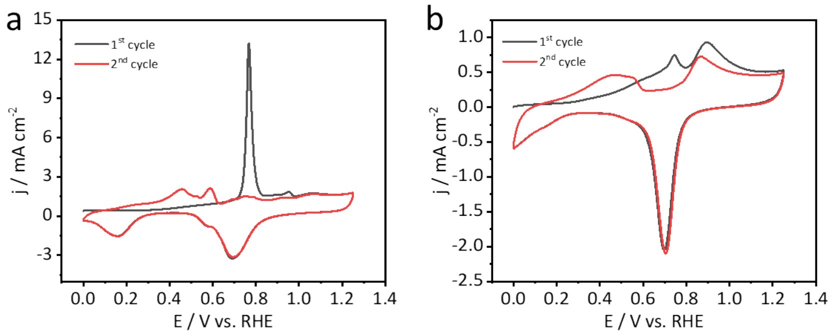

Figure S31. CO stripping voltammograms of a) Pd and b) PdCuZn NSs in 1.0 M KOH (aq) with a sweep rate of 20 mV s^{-1}.

Table S1. Element composition of as-prepared Pd-based alloys from ICP-OES measurements.

Sample	Element	Atomic percentage/%	Mass percentage/%
$Pd_{3.96}Cu_{1.06}Zn_{0.98}$	Pd	66.00	88.27
	Cu	17.67	6.33
	Zn	16.33	5.40
$Pd_{4.02}Cu_{2.36}Zn_{1.03}$	Pd	54.25	70.91
	Cu	31.85	24.44
	Zn	13.90	4.65
$Pd_{3.98}Cu_{2.05}Zn_{0.54}$	Pd	60.58	71.92
	Cu	31.20	22.12
	Zn	8.22	5.96
$Pd_{4.07}Cu_{1.93}Zn_{0.66}$	Pd	61.11	72.35
	Cu	28.98	20.48
	Zn	9.91	7.17
$Pd_{3.99}Cu_{1.96}Zn$	Pd	57.39	69.08
	Cu	28.23	20.29
	Zn	14.38	10.63
$Pd_{1.99}Cu$	Pd	66.51	76.88
	Cu	33.49	23.12
$Pd_{4.13}Zn$	Pd	80.54	87.08
	Zn	19.46	12.92

Table S2. EXAFS fitting structural parameters of PdCuZn.

Edge	Path	CN	R/Å	σ^{2}/Å^{2}	ΔE/eV	R-factor
Pd	Pd-Cu	1.89	2.58	0.006	9.43	0.011
	Pd-M	8.64	2.67	0.011	3.11	
Cu	Cu-Cu	1.57	2.55	0.009	2.40	0.008
	Cu-M	7.84	2.68	0.006	5.44	
Zn	Zn-Cu	1.09	2.57	0.014	5.80	0.016
	Zn-M	6.79	2.65	0.012	1.92	

CN, coordination number; R, bonding length; σ^2, Debye-Waller factor; M, Zn/Pd; ΔE, energy shift; R-factor is used to value the goodness of the fitting.

Table S3. Summary of the performances of Pd-based electrocatalysts for electrochemical CO_2RR in flow cell.

Electrocatalysts	Electrolytes	Potentials / V vs. RHE	FE_{CO} / %	j_{CO} / mA cm^{-2}	Stability	References
C-Bi_6Pd_{94}-SAA	1.0 M KOH	−0.48 V	84.7	~173.3	−0.40 V for 20 hours	*Appl. Catal. B* **2021**, *289*, 119783
Pd/PdO H-NWs	1.0 M KOH	−0.7	95.6	127	−0.8 V for 12 hours	*J. Energy Chem*, **2022**, *70*, 407−413
PdIn@In_2O_3	1.0 M KOH	−0.9	92.1		−0.9 V for 24 hours	*ACS Nano* **2022**, *16*, 6185−6196
Cu5Pd5	1.0 M KOH	−0.7	88	132		*Adv. Energy Sustainability Res.* **2022**, *3*, 2200075
Pd_1Cu_1	1.0 M KOH	−0.68	96	~221	−0.68 V for 17 hours	*Sep. Purif. Technol.*, **2023**, *320*, 124186
$Cu_{100}Bi$	1.0 M KOH	−0.76	86.6	259.8		*Appl. Catal. B*, **2022**, *317*, 121650
Ordered CuPd	1.0 M KOH	−0.55	80	49.2		*J. Am. Chem. Soc.* **2017**, *139*, 47−50
Cs_2PdBr_6 NCs	1.0 M KOH	−0.7	78		−0.7 V for ~9 hours	*Energy Environ. Mater.*, **2023**, *6*, e12411

$Cu_{97}Sn_3$(F)	1.0 M KOH	−0.45	87	87	−0.75 V for 20 hours	*Nat Commun.*, **2021**, *12*, 1449
CoPc©Fe–N–C	0.5 M KOH	−0.6	＞90	100	−0.55 V for 20 hours	*Adv. Materials.*, **2019**, *31*, 1903470
$Ag_{70}Cu_{30}$	1.0 M KOH	−0.75	~80	~92	−1.0 V for 20 hours	*Small* **2023**, *19*, 2207242
PdCuZn NSs	1.0 M KOH	−0.35<br>−0.71	96<br>80.21	26.62<br>143.26	−0.35 V for 100 hours	This work

References

(1) Yang, Q.; Shi, L.; Yu, B.; Xu, J.; Wei, C.; Wang, Y.; Chen, H. Facile synthesis of ultrathin Pt-Pd nanosheets for enhanced formic acid oxidation and oxygen reduction reaction. *J. Mater. Chem. A* **2019**, *7*, 18846−18851.

(2) Kresse, G.; Joubert, D. From ultrasoft pseudopotentials to the projector augmented-wave method. *Phys. Rev. B* **1999**, *59*, 1758.

(3) Perdew, J. P.; Burke, K.; Ernzerhof, M. Generalized gradient approximation made simple. *Phys. Rev. Lett.* **1996**, *77*, 3865.

(4) Hansen, H. A.; Varley, J. B.; Peterson, A. A.; Norskov, J. K. Understanding Trends in the Electrocatalytic Activity of Metals and Enzymes for CO_2 Reduction to CO. *J. Phys. Chem. Lett.* **2013**, *4*, 388−392.

(5) Karamad, M.; Hansen, H. A.; Rossmeisl, J.; Nørskov, J. K. Mechanistic Pathway in the Electrochemical Reduction of CO_2 on RuO_2. *ACS Catal.* **2015**, *5*, 4075−4081.

(6) Peterson, A. A.; Abild-Pedersen, F.; Studt, F.; Rossmeisl, J.; Norskov, J. K. How copper catalyzes the electroreduction of carbon dioxide into hydrocarbon fuels. *Energy Environ. Sci.* **2010**, *3*, 1311−1315.
